# A conformational switch driven by phosphorylation regulates the activity of the evolutionarily conserved SNARE Ykt6

**DOI:** 10.1073/pnas.2016730118

**Published:** 2021-03-15

**Authors:** Kaitlyn McGrath, Shivani Agarwal, Marco Tonelli, Mykola Dergai, Anthony L. Gaeta, Andrew K. Shum, Jessica Lacoste, Yongbo Zhang, Wenyu Wen, Daayun Chung, Grant Wiersum, Aishwarya Shevade, Sofia Zaichick, Damian B. van Rossum, Ludmilla Shuvalova, Jeffrey N. Savas, Sergei Kuchin, Mikko Taipale, Kim A. Caldwell, Guy A. Caldwell, Dirk Fasshauer, Gabriela Caraveo

**Affiliations:** ^a^Department of Neurology, Feinberg School of Medicine, Northwestern University, Chicago, IL 60611;; ^b^The National Magnetic Resonance Facility at Madison, University of Wisconsin, Madison, WI 53706;; ^c^Department of Fundamental Neurosciences, University of Lausanne, 1005 Lausanne, Switzerland;; ^d^Department of Biological Sciences, The University of Alabama, Tuscaloosa, AL 35401;; ^e^Department of Molecular Genetics, University of Toronto, Toronto, ON M5S 1A8, Canada;; ^f^Integrated Molecular Structure Education and Research Center, Department of Chemistry, Northwestern University, Evanston, IL 60208;; ^g^Institutes of Biomedical Sciences, School of Basic Medical Sciences, Fudan University, Shanghai 200032, China;; ^h^Center for Structural Genomics of Infectious Diseases, Northwestern University, Chicago, IL 60611;; ^i^Department of Biological Sciences, University of Wisconsin, Milwaukee, WI 53211;; ^j^Division of Experimental Pathology, Department of Pathology, Penn State College of Medicine, Hershey, PA 17033;; ^k^The Jake Gittlen Laboratories for Cancer Research, Penn State College of Medicine, Hershey, PA 17033;; ^l^Donnelly Centre for Cellular and Biomolecular Research, University of Toronto, Toronto, ON M5S 3E1, Canada;; ^m^Department of Neurology, Center for Neurodegeneration and Experimental Therapeutics, Nathan Shock Center of Excellence in the Basic Biology of Aging, University of Alabama at Birmingham School of Medicine, Birmingham, AL 35249;; ^n^Department of Neurobiology, Center for Neurodegeneration and Experimental Therapeutics, Nathan Shock Center of Excellence in the Basic Biology of Aging, University of Alabama at Birmingham School of Medicine, Birmingham, AL 35249

**Keywords:** SNARE, calcineurin, Parkinson’s disease, conformation, Ykt6

## Abstract

Ykt6 is a soluble *N*-ethylmaleimide sensitive factor activating protein receptor (SNARE) critically involved in diverse vesicular fusion pathways. While most SNAREs rely on transmembrane domains for their activity, Ykt6 dynamically cycles between the cytosol and membrane-bound compartments where it is active. The mechanism that regulates these transitions and allows Ykt6 to achieve specificity toward vesicular pathways is unknown. Using a Parkinson’s disease (PD) model, we found that Ykt6 is phosphorylated at an evolutionarily conserved site which is regulated by Ca^2+^ signaling. Through a multidisciplinary approach, we show that phosphorylation triggers a conformational change that allows Ykt6 to switch from a closed cytosolic to an open membrane-bound form. In the phosphorylated open form, the spectrum of protein interactions changes, leading to defects in both the secretory and autophagy pathways, enhancing toxicity in PD models. Our studies reveal a mechanism by which Ykt6 conformation and activity are regulated with potential implications for PD.

## Significance

Ykt6 is a conserved SNARE that plays critical roles along multiple vesicular pathways. To achieve its function, Ykt6 cycles between the cytosol and membrane-bound compartments through reversible lipidation. The mechanism that regulates these transitions is unknown. Ykt6 function is disrupted by α-synuclein, a protein critically implicated in synucleinopathies such as Parkinson’s Disease. Through a multidisciplinary approach, we report that phosphorylation regulated by Ca^2+^ signaling drives a conformational change that allows Ykt6 to switch from a closed cytosolic to an open membrane-bound form. Phosphorylation is also a critical determinant for Ykt6 protein interactions with functional consequences in the secretory and autophagy pathways under normal and α-synuclein conditions. This work provides a mechanistic insight into Ykt6 regulation with therapeutic implications for synucleinopathies.

Membrane fusion represents the final stage in vesicle trafficking and is largely driven by the soluble *N*-ethylmeleimide sensitive factor activating protein receptor (SNARE) proteins, which contribute to the specificity of the trafficking event (1, 2). Ykt6 is an essential R-SNARE and one of the most highly conserved SNAREs in all eukaryotes (3, 4). Ykt6 plays a key role in numerous vesicular transport pathways in yeast and in mammalian cells: 1) the secretory pathways, which are endoplasmic reticulum (ER) to the Golgi apparatus (Golgi), intra-Golgi, Golgi–ER retrieval pathways, and in the constitutive transport from the Golgi to the plasma membrane (3, 5, 6); 2) the endocytic pathways, which are between the Golgi and the vacuole/lysosome (7, 8) and endosomes to exosomes (9); and 3) the macroautophagy pathway (hereafter referred to as autophagy) (10–12). To date, the mechanism(s) underlying Ykt6 recruitment to distinct vesicular pathways remains unresolved.

Unlike most SNAREs, Ykt6 contains a C-terminal lipid anchor motif which can be reversibly either palmitoylated (7, 13, 14) or geranylgeranylated (15) at cysteine 194 and permanently farnesylated at cysteine 195 (16). The reversible nature of palmitoylation and/or geranylgeranylation has been proposed as a mechanism to regulate Ykt6 membrane association, allowing it to cycle between the cytosol and membrane-bound compartments (14, 17). While lipid modifications are crucial to regulate Ykt6 membrane association, NMR and crystallography studies demonstrated that activation of Ykt6 is far more complex (18). When in the cytosol, Ykt6 forms a closed conformation whereby the N-terminal regulatory longin domain folds back onto the SNARE domain stabilized by hydrophobic interactions (18–20). Deletion of the longin domain, or substitution of a hydrophobic residue to a negatively charged one within the Ykt6 longin domain (F42E), causes the longin domain to dissociate from the SNARE domain and relocalize Ykt6 to both the plasma membrane and Golgi (14, 18). These data have led to a model whereby, for Ykt6 to be active and membrane associated, the longin and SNARE domains must separate to facilitate the open conformation. Once in the open conformation, Ykt6 can be palmitoylated, providing its membrane stability and interactions with its SNARE partners (14, 19). Whether this is the molecular mechanism that activates Ykt6, as well as the in vivo physiological triggers, remains to be shown.

**Fig. 1.**
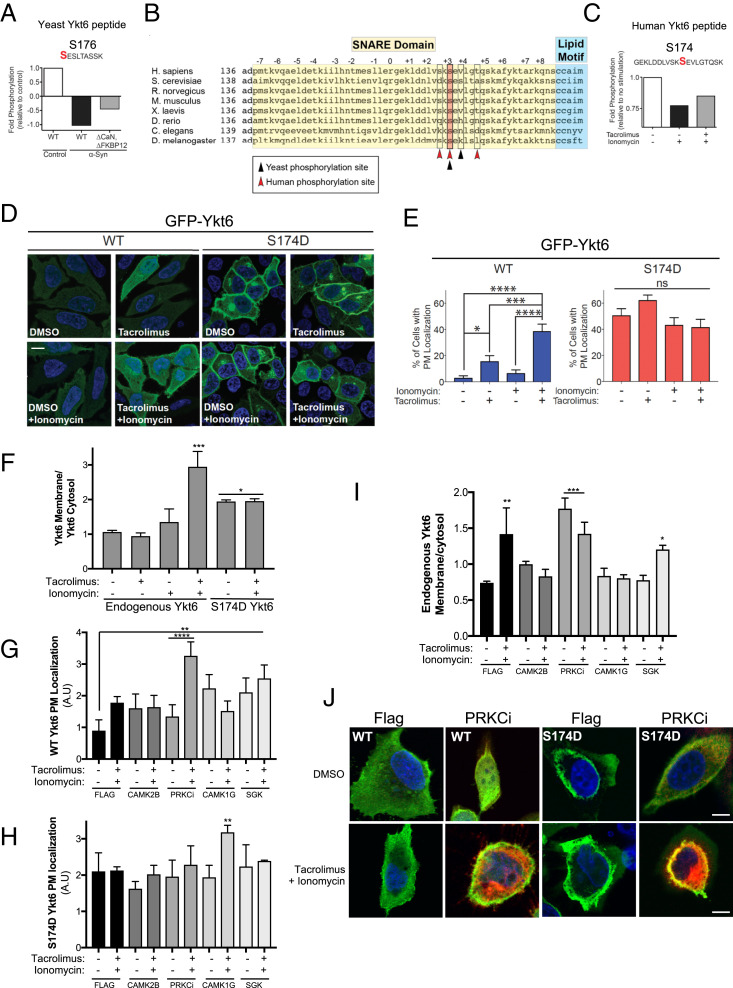
Evolutionarily conserved phosphorylation site within human Ykt6 SNARE domain (S174) is sensitive to calcineurin, evolutionarily conserved in yeast and in humans, and is a critical determinant for its intracellular localization. (*A*) Fold phosphorylation of the indicated peptide from endogenous yeast Ykt6 detected by shotgun phosphoproteomics after correction for protein abundance from control yeast cells and yeast cells with high levels of Ca^2+^ (driven by overexpression of α-syn) with either WT or knockout for calcineurin (∆CaN) and knockout for the modulator of calcineurin (∆FKBP12). The identified phosphorylation sites are highlighted in red. Data from triplicate samples were pulled together for illustrated analysis (28). Data from ref. 27. (*B*) Alignment of Ykt6 sequences across species. Highlighted in pink is the identified phosphorylation site conserved across evolution. Arrows depicted represent Ykt6 zero-layer arginine (white) and additional CaN-sensitive sites identified in phosphoproteomic screen (red is human, black is yeast). (*C*) Fold phosphorylation of the indicated human Ykt6 peptide from HEK293T cells as detected by iTRAQ MS. Prior to GFP-Ykt6 immunoprecipitation, cells were treated for 30 min with the Ca^2+^ ionophore ionomycin (1 μM) and cotreated with calcineurin-specific inhibitor tacrolimus (1 μM). (*D*) Representative IF images of transiently transfected HeLa cells with GFP-tagged wild-type (WT) or phosphomimetic mutant of Ykt6 (S174D). Cells were treated with ionomycin (1 μM) and/or tacrolimus (1 μM) for 30 min. Nuclei (blue) are stained with DAPI. (Scale bar, 10 μm.) (*E*) Quantification of cells with GFP plasma membrane localization as shown in *A*. *n* = 3; *P < 0.05, ***P < 0.001, ****P < 0.0001. One-way ANOVA, uncorrected Fisher’s least significance difference (LSD) test. (*F*) Quantification of Ykt6 on membrane fractions (Na^+^K+)/Ykt6 on cytosol fractions (tubulin) from Western blots after cell fractionation (see Dataset S1); *n* = 3. (*G*, *H*) Quantification of GFP-WT Ykt6 (*G*) or GFP-S174D Ykt6 (*H*) on PM fraction (see *Materials and Methods* for details) of HEK293T cotransfected with the indicated Flag-tagged kinases. (*I*) Quantification of endogenous Ykt6 membrane (Na^+^K+)/Ykt6 cytosol (tubulin) fraction from Western blots after cell fractionation of HEK293T cells transfected with the indicated Flag-tagged kinases; *n* = 3 (see *SI Appendix*, Fig. S5*D*). Stats for (*F*–*I*) **P < 0.05, ***P < 0.001, ****P < 0.0001. One-way ANOVA, uncorrected Fisher’s LSD test. All comparisons to dimethyl sulfoxide (dmso) control of Flag-transfected dmso. (*J*) 40× confocal images after IF for YKT6 (green) and FLAG-tagged candidate kinases (red) of HEK293T cells cotransfected with GFP-WT-Ykt6 and the indicated Flag-tagged kinase. Single Z-frame images were channel merged and background subtracted before processed for quantitative analysis. Individual representative cells were chosen from each condition to better present changes in intracellular localization of YKT6. (Scale bar, 10 μm.) *n* = 3; ∼600 cells total.

Misfolding of α-synuclein (α-syn) is the pathological hallmark of both familial and sporadic Parkinson’s disease (PD) (21–24). α-Syn triggers high levels of cytosolic Ca^2+^ and the activation of calcineurin (CaN), a Ca^2+^-dependent phosphatase (25, 26). Using an unbiased phosphoproteomic approach in a yeast model for α-syn toxicity, we previously found endogenous Ykt6 phosphorylated and regulated by CaN (27) (Fig. 1*A*). Ykt6 itself has been implicated in α-syn pathobiology as Ykt6 overexpression overcomes the secretory trafficking deficits caused by α-syn and protects against cell death (28, 29). Whether Ykt6 regulation by phosphorylation contributes to Ykt6 deficits under α-syn toxicity remains to be determined.

Here, we report that phosphorylation at the evolutionarily conserved site S174 within the Ykt6 SNARE domain drives an intramolecular rearrangement mediating the conversion from a closed cytosolic to an open membrane-bound state. Using an unbiased high-content kinase screening assay, we found that protein kinase C iota type (PRKCi) regulates Ykt6 phosphorylation and membrane association. Furthermore, we demonstrate that phosphorylation is sensitive to CaN and a key determinant of the binding affinity and specificity for various protein interactions. Phospho-dependent interactions of Ykt6 have functional consequences in two cellular activities in which Ykt6 has been shown to play a role: the secretory and autophagy pathways. Moreover, we show the biological importance of the evolutionarily conserved phosphosite under physiological and α-syn toxic conditions in yeast and *Caenorhabditis elegans* models of PD. Taken together, our results provide a mechanistic insight into the regulation of Ykt6 and its cellular activities with implications for PD, wherein malfunctions in Ykt6, autophagy, and the secretory pathway have been attributed (28–30).

## Results

### A Conserved Phosphorylation Site within Ykt6 SNARE Domain Is Sensitive to Calcineurin and Is a Key Determinant for Ykt6 Intracellular Localization.

We previously established a central role for the 12 kDa *cis–trans* proline isomerase FK506-binding protein (FKBP12) and CaN in α-syn toxicity (27). Specifically, we have found that α-syn leads to a pathological increase in cytosolic Ca^2+^ and a subsequent increase in CaN/FKBP12 activity, which leads to cell death (25, 27). To identify substrates dephosphorylated by CaN/FKBP12 associated to this toxic response, we undertook an unbiased phosphoproteomic screen in a yeast model of α-syn toxicity (27). We focused on endogenous peptides hypophosphorylated in the presence of α-syn, which became increasingly phosphorylated in CaN/FKBP12 knockout yeast cells. With these criteria, we retrieved two phosphosites from Ykt6, S176, and S178 (27) (Fig. 1 *A* and *B* and *SI Appendix*, Fig. S1*A*). Both phosphosites lie within the yeast Ykt6 SNARE domain, but only S176 is highly conserved from fungi to animals (Fig. 1*B* and *SI Appendix*, Fig. S1*B*). Thus, CaN regulates endogenous Ykt6 at the evolutionarily conserved phosphosite S176 in yeast.

Next, we investigated whether human Ykt6 is similarly phosphorylated and sensitive to CaN phosphatase activity. We transfected human embryonic kidney (HEK293T) cells with human Ykt6 N-terminally tagged with the green fluorescent protein (GFP) for three important reasons: 1) cell lines amenable to scale up for mass spectrometry (MS) such as HEK-293T or HeLa cell express very little endogenous Ykt6 (*SI Appendix*, Fig. S1*C*); 2) to maintain similar efficiency of Ykt6 immmunoprecipitate (IP) under the diverse Ca^2+^-stimulating conditions by using the tag as a bait; and 3) N-terminal tagging of Ykt6 does not affect its function as shown by others (14). To mimic the high CaN activity from α-syn expression in yeast, cells were treated with the Ca^2+^ ionophore ionomycin to trigger a rapid increase in cytosolic Ca^2+^ and hence activate CaN. In parallel, cells were cotreated with ionomycin and the CaN-specific inhibitor tacrolimus to block CaN phosphatase activity. Using the GFP tag, Ykt6 was pulled down from each condition and subjected to MS by isobaric tag for relative and absolute quantitation (iTRAQ, Dataset S1). We retrieved three phosphosites in human Ykt6 (S172, S174, and T179) whose phosphorylation was decreased in the presence of ionomycin and was partially restored with the addition of tacrolimus (Fig. 1 *B* and *C* and *SI Appendix*, Fig. S1*D*). Importantly, S174 is homologous to the conserved site S176 retrieved from the yeast phosphoproteomic screen (Fig. 1*B* and *SI Appendix*, Fig. S1*B*). While all human phosphosites were highly conserved in animals (*SI Appendix*, Fig. S1*B*), only S174 was conserved throughout the phylogenic tree (Fig. 1*B*).

To examine if the evolutionarily conserved CaN-sensitive phosphosite S174 is physiologically relevant in mammalian cells, we expressed N-terminal GFP fusions of human wild-type (WT) Ykt6 or the phosphomimetic (S174D) mutant of Ykt6 in HeLa cells. While WT Ykt6 was mainly present in the cytosol, the phosphomimetic mutant showed a Golgi-like and plasma membrane localization (Fig. 1 *D* and *E*). To investigate whether the plasma membrane localization of the S174D mutant was dependent on CaN, we took a pharmacological approach. HeLa cells transfected with WT Ykt6 were Ca^2+^ stimulated with ionomycin and/or treated with the CaN-specific inhibitor tacrolimus. Indeed, inhibition of CaN caused an increase in WT Ykt6 plasma membrane localization (Fig. 1 *D* and *E*). Inhibition of CaN under Ca^2+^ stimulation with ionomycin further increased WT Ykt6 plasma membrane localization, suggesting that phosphorylation at this site is also Ca^2+^ dependent. We did not observe any significant changes in intracellular localization in cells transfected with GFP alone or the phosphomimetic mutant under the same pharmacological treatment (Fig. 1 *D* and *E* and *SI Appendix*, Fig. S1 *E* and *F*). Importantly, cotreatment of ionomycin and tacrolimus also changed the intracellular localization of endogenous Ykt6 from the cytosol to membrane-bound compartments assayed by cell fractionation (Fig. 1*F* and *SI Appendix*, Fig. S1*I*).

To identify the kinase(s) responsible for Ykt6 phosphorylation and cytosol versus membrane localization, we conducted a high-content kinase screen. From the 355 unique protein kinases available in a human kinome Flag tagged Gateway library, we screened a total of 300 clones after DNA verification. Each clone was co-overexpressed with GFP-WT Ykt6 into HeLa cells on a 96-well plate, stained with wheat germ agglutinin (WGA) as a Golgi and plasma membrane marker, and subjected to high-content imaging. Positive hits were considered as following: signal/background = positive control (average colocalization signal intensity between WGA and S174D)/negative control (average colocalization signal intensity between WGA and WT Ykt6 no kinase) > fivefold. Under these criteria, we retrieved 30 putative kinase hits that, when overexpressed, conferred GFP-WT Ykt6 Golgi-like and/or plasma membrane (PM) (*SI Appendix*, Fig. S1*G*). After validation, only four kinases showed consistent PM and Golgi-like localization for GFP-WT Ykt6 (Fig. 1*G* and *SI Appendix*, Fig. S1*H*). Of these, only PRKCi increased the membrane association of endogenous Ykt6 assayed by cellular fractionation and did not change the PM localization of GFP-S174D Ykt6 under normal and Ca^2+^-stimulating conditions (Fig. 1 *H* and *I* and *SI Appendix*, Fig. S5*D*). Ca^2+^ stimulation and CaN inhibition in the presence of PRKCi did not change membrane association of endogenous Ykt6 relative to no pharmacological treatment assayed by cellular fractionation (Fig. 1*I*). However, Ca^2+^ stimulation and CaN inhibition in the presence of PRKCi did increase PM localization of GFP-WT Ykt6 by immunofluorescence (IF) (Fig. 1 *G* and *J*). These data are consistent with our previous result whereby inhibition of CaN under Ca^2+^ stimulation increased PM association of GFP-WT Ykt6 in the absence of overexpressed kinase (Fig. 1 *D* and *E*). PRKCi is a noncanonical protein kinase C which is recruited to the ER–Golgi compartment (ERGIC organelle) with a role in microtubule dynamics in the early secretory pathway. Together, these results indicate that the phosphorylation site S174 we identified within the endogenous Ykt6 SNARE domain is evolutionarily conserved, physiologically regulated by PRKCi, Ca^2+^, and CaN, and is a critical determinant for Ykt6 membrane localization.

### Evolutionarily Conserved Phosphorylation Site within the SNARE Domain (S174) Regulates Ykt6 Open Conformation.

**Fig. 2.**
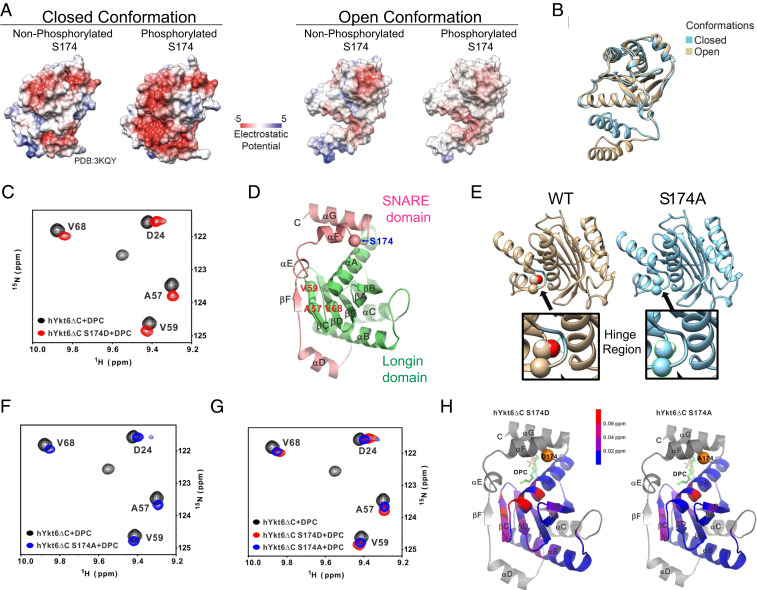
Calcineurin-sensitive evolutionarily conserved phosphorylation site S174 in Ykt6 SNARE domain regulates its conformation. (*A*) Electrostatic potential computed and projected at the surface representation of both open and closed conformation in the presence and absence of phosphorylation at the evolutionarily conserved site S174. (*B*) Ykt6 secondary structure depicting the open (brown) and closed (blue) conformation. (*C*) ^15^*N*-^1^H TROSY of 0.4 mM 25 kDa Ykt6 at 600 MHz. WT Ykt6 (black) versus S174D (red) in the presence of DPC. (*D*) Ykt6 crystal structure. Longin domain (green) and the SNARE domain (pink); in red are the amino acids where a chemical shift was detected and in blue is the S174 phosphorylation site. (*E*) Structural model showing the critical positioning of S174 in the hinge loop and how the alanine replacement loosens the interaction of between the SNARE and the longin domains. The hinge region is enlarged for both the WT and phosphoablative S174A Ykt6. (*F*) ^15^*N*-^1^H TROSY of 0.4 mM 25 kDa Ykt6 at 600 MHz. WT Ykt6 (black) and S174A (blue) in the presence of DPC. (*G*) ^15^*N*-^1^H TROSY of 0.4 mM 25 kDa Ykt6 at 600 MHz. WT Ykt6 (black) and phosphomimetic S174D (red) and phosphoablative S174A (blue) in the presence of DPC (*J*). (*H*) Ykt6 crystal structure denoting chemical shifts detected throughout the protein; below 0.02 ppm is not significant.

Previous work demonstrated that cytosolic Ykt6 exists in a closed conformation whereby the longin and the SNARE domains are in contact through hydrophobic interactions (19). We asked whether phosphorylation at S174 could disrupt the hydrophobic interactions between the SNARE and longin domains, therefore triggering Ykt6 open conformation. We first took a bioinformatics-based structural approach. It is known that the Ykt6 closed conformation is stabilized by an extended network of hydrophobic amino acids in the longin domain (such as F34 and F42) and in the SNARE domain (such as V171) which, when individually mutated to a glutamate, leads to an open conformation (19). The S174 site is located at the end of the αF helix in the SNARE domain, which makes it accessible to phosphatases and kinases (*SI Appendix*, Fig. S2*A*). When we modeled the phosphorylated form of this residue using the available Ykt6 closed conformation (19), the S174 phosphate group could be placed into the structure without introducing unresolvable clashes of atoms due to spatial constraints (*SI Appendix*, Fig. S2*B*). Therefore, we asked if phosphorylation could contribute to the electrostatic repulsion between the longin and SNARE domains and influence Ykt6 conformation. We noted a conserved patch of negatively charged residues in close structural proximity to the CaN-dependent phosphorylation sites in the αF helix SNARE domain and at the longin domain around a short helix turn (αE) (*SI Appendix*, Fig. S2*C*). To investigate whether the highly conserved negatively charged patch might contribute to an electrostatic repulsion between the helices, we slightly adjusted the Ykt6 closed conformation by fine-tuning the torsion angles of the loop region, residues 162 to 164. This allows visualization of the compact electrostatic potential in this region that would otherwise be visible only at the surface. After such adjustment, we noted a significant decrease in electrostatic potential which could drive electrostatic repulsion between these regions and displacement of the longin and SNARE domain (Fig. 2 *A* and *B* and *SI Appendix*, Fig. S2*D*). These data suggest that electrostatic repulsion acting upon longer distances can prevent short-range hydrophobic binding favoring Ykt6 open conformation.

To directly examine the effect of S174 phosphorylation on Ykt6 conformation, we used NMR in solution. Recombinant human WT Ykt6 and S174D mutant proteins lacking the lipid binding motif (ΔC) were expressed and purified from bacteria. We deleted the lipid binding motif to avoid protein aggregation (*SI Appendix*, Fig. S2*E*). To mimic farnesylation and stabilize the protein, we exogenously added dodecylphosphocholine (DPC) to the purified protein preparations as other groups have reported (19, 20). Indeed, in the absence of DPC, there is a large dynamic range in the intensity of the peaks in the ^15^*N*-HSQC spectrum of WT Ytk6, with some strong peaks and many broad and weak peaks, an indication of a dynamic and not well-defined single conformation (*SI Appendix*, Fig. S2 *F*–*H*). After adding DPC, the quality of the ^15^*N*-HSQC spectrum improved significantly, with new peaks appearing and older ones becoming sharper, yielding a more stable Ytk6 structure (*SI Appendix*, Fig. S2 *F*–*H*).

When we compared the ^1^H ^15^*N*-HSQC spectra of hYkt6ΔC WT and S174D in the presence of saturating amounts of DPC, we detected a shift in some peaks while others become broader and disappeared (Fig. 2 *C* and *D* and *SI Appendix*, Fig. S2 *I*–*K*). Peak shifting indicates conformational perturbation in the mutant, while peak broadening and disappearance suggests that the structure of the mutant may not be as well defined as that of the WT protein. S174D resulted in chemical shift changes to a large number of residues in the longin domain, at two regions: the top αA in direct contact with the S174-containing α-helix of SNARE domain and the central β-strands, β-C (A57 and V59) and β-D (H67,V68), which make backbone hydrogen bonds with the small β-F strand of the SNARE domain to form a sheet and a close conformation (Fig. 2 *C* and *D*). We observed chemical shifts in the same amino acids in the absence of DPC except of larger magnitude (*SI Appendix*, Fig. S3*A*), corroborating the conformational perturbation in the mutant.

After careful inspection of Ykt6 crystal structure, we noticed that S174 is involved in two hydrogen bonds with the backbone of the two neighbors placed in the hinge region/loop (V176 and L177), which might facilitate the closed conformation (Fig. 2*E*). In a phosphorylation-deficient mutant such as S174A, the structure is energetically penalized whereby two nitrogen atoms are not involved in hydrogen bonds and are isolated from water molecules as there is not enough space to bring them in close proximity, potentially shifting the equilibrium toward the open conformation (Fig. 2*E*). To directly examine this, we analyzed the S174A structure by NMR. Indeed, the S174A caused significant chemical shift changes relative to the WT, although to a smaller scale compared to the S174D mutant (Fig. 2 *F*–*H* and *SI Appendix*, Fig. S3 *B*–*E*). More peaks disappeared from the spectrum of the S174D mutant compared to S174A (*SI Appendix*, Fig. S3 *B*–*E*), which suggests that perturbations at S174 can cause conformational change, destabilizing the Ykt6 structure with S174D being more open than S174A.

To directly examine the effect of S174 phosphorylation on Ykt6 conformation biochemically, we used partial proteolysis with trypsin since open conformations tend to be more susceptible to proteolytic cleavage than closed conformations. GFP-WT Ykt6 and phospho-mutants were purified from HEK293T cells using GFP and incubated with different concentrations of trypsin for 1 h (*SI Appendix*, Fig. S3*F*). While the WT Ykt6 displayed a proteolytic fragment with 10 ng of trypsin, both phospho-mutants displayed their first proteolytic cleavage product with only 5 ng of trypsin supporting an open conformation relative to the WT (*SI Appendix*, Fig. S3*F*).

To further support that phosphorylation within the Ykt6 SNARE domain can prevent the interaction between the SNARE and longin domains, we used a fluorescence complementation approach in HeLa cells. One vector contained the N-terminal half of Venus fused to the Ykt6 longin domain, while the second vector contained the C-terminal half of Venus fused to the Ykt6 SNARE domain of WT, S174A, or S174D mutants. All constructs lacked the lipid anchor motif CCAIM to avoid complications because of the inherent differences in their subcellular localization. If both phospho-mutants can trigger the open conformation, albeit for different reasons, we expected both phospho-mutants to have less complementation between the split Venus domains compared to WT Ykt6 and consequently less Venus fluorescence. Expression of a single split Venus construct had little to no background fluorescence (*SI Appendix*, Fig. S3*G*); however, coexpression of the WT SNARE domain with the longin domain generated a robust Venus fluorescent signal indicative of a strong interaction between the longin and SNARE domains. However, the use of either phospho-mutant SNARE domain yielded less Venus fluorescence complementation compared to the WT Ykt6 SNARE domain (*SI Appendix*, Fig. S3*H*).

Taken together, structural modeling, NMR, biochemical, and cellular experiments support a critical role of the evolutionarily conserved site S174 in regulating Ykt6 open conformation. Mimicking phosphorylation at S174 (S174D mutant) destabilizes the hydrophobic interaction between the longin and SNARE domains, promoting the open conformation. While the S174A mutant caused similar structural constraints, albeit to a lesser extent, it provided an ideal tool to dissect the contributions of the open conformation on protein interactions in the absence of phosphorylation, a scenario representing CaN activation.

### Ykt6 Open Conformation Is a Key Determinant for Its Membrane Association.

**Fig. 3.**
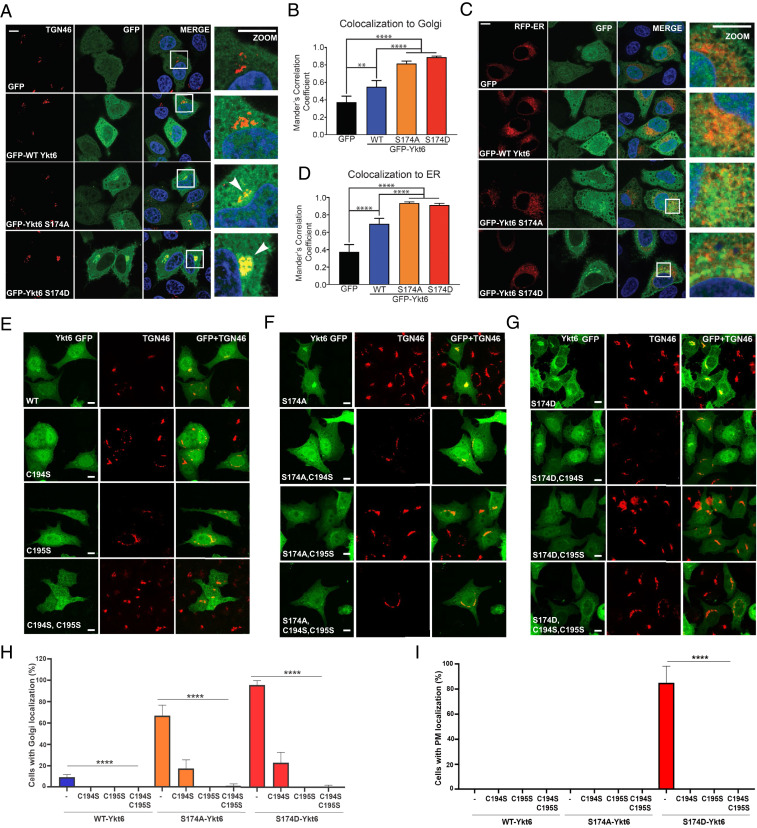
Open conformation regulated by the calcineurin-sensitive evolutionarily conserved site S174 within Ykt6 SNARE domain is a critical determinant for its membrane localization. (*A*) Representative IF images of transiently transfected HeLa cells with GFP, GFP-tagged WT, or phospho-mutants of Ykt6 and immunostained for TGN-46, a *trans*-Golgi marker. Nuclei (blue) are stained with DAPI. (*B*) Colocalization analysis based on Mander’s correlation coefficient between TGN-46 and Ykt6. (Scale bars in *D*–*F*, 10 μm.) *n* = 3; **P < 0.01, ****P < 0.0001. One-way ANOVA, Tukey’s test. (*C*) Representative IF images of transiently cotransfected HeLa cells expressing GFP, GFP-tagged WT, or indicated phospho-mutants of Ykt6 along with mCherry-Sec61 (ER-RFP) to delineate the ER. Nuclei (blue) are stained with DAPI. (*D*) Same as in *B* but between ER and Ykt6. (*E*–*G*) Representative IF images of HeLa cells transiently transfected with either the single farnesylation (C195S), palmitoylation (C194S), or the double lipid (C194S_C195S) deficient mutants in Ykt6 WT (*E*), S174A (*F*), or S174D (*G*) background. Cells were immunostained for TGN-46, a *trans*-Golgi marker. (*H*, *I*) Colocalization analysis based on Mander’s correlation coefficient for TGN-46 depicting the percentage of cells with Ykt6 localized in either *trans*-Golgi (*H*) PM (*I*). *n* = 150 cells (two biological replicates) and ****P < 0.0001. One-way ANOVA, Tukey’s test. (Scale bar, 10 μm.)

To examine if the open conformation leads to a specific subcellular localization, we expressed GFP-WT Ykt6, S174A, or S174D mutants in HeLa cells. Importantly, we found no difference in their transient expression levels (*SI Appendix*, Fig. S4 *A* and *B*). While all the constructs were present in the cytosol, the phospho-mutants showed an additional colocalization with the *trans*-Golgi marker TGN46 (Fig. 3 *A* and *B*) and a less obvious colocalization with the ER marker Sec61 (Fig. 3 *C* and *D* and *SI Appendix*, Fig. S4*C*). Furthermore, the S174D mutant also localized to the PM (Figs. 1 *D* and *E* and 3 *A* and *C*). Consistent with these results, cell fractionation demonstrated that the phospho-mutants had increased membrane association relative to the WT Ykt6, with phosphomimetic mutant S174D showing the highest (*SI Appendix*, Fig. S4 *D* and *E*).

Lipid modifications at the C-terminal cysteine residues of Ykt6 play an important role in the interconversion of the cytosolic to membrane-bound pools, hence Ykt6 function. There is irreversible farnesylation at Cys-195 (14) and reversible palmitoylation or gernaylgeranylation at Cys-194 (15, 14). Farnesylation at Cys-195 has been shown to keep the protein in the closed conformation in the cytosol (19), whereas the additional geranylgeranyl or palmitoyl group at Cys-194 allows Ykt6 to be stably inserted into membranes (14, 19). To investigate how the open conformation at S174 could influence Ykt6 membrane association, we mutated both cysteines individually and in combination. Abolishment of de novo palmitoylation or geranylgeranylation (C194S) reduced the Golgi localization in both phospho-mutants to about 20% (Fig. 3 *F*–*H*). However, abolishment of the permanent farnesylation (C195S) completely eliminated the Golgi localization of the WT and phospho-mutants (Fig. 3 *E*–*H*). The PM localization of the S174D mutant was eliminated with both C194S and C195S (Fig. 3 *G* and *I*). Together, these results suggest that triggering the open conformation (by phosphorylation or other structural constraints) at the evolutionary conserved site S174 enables Ykt6 lipid modifications, farnesylation being more critically involved for its membrane association.

### Phosphorylation at S174 Regulates Ykt6 Specificity and Affinity to Its Binding Partners.

**Fig. 4.**
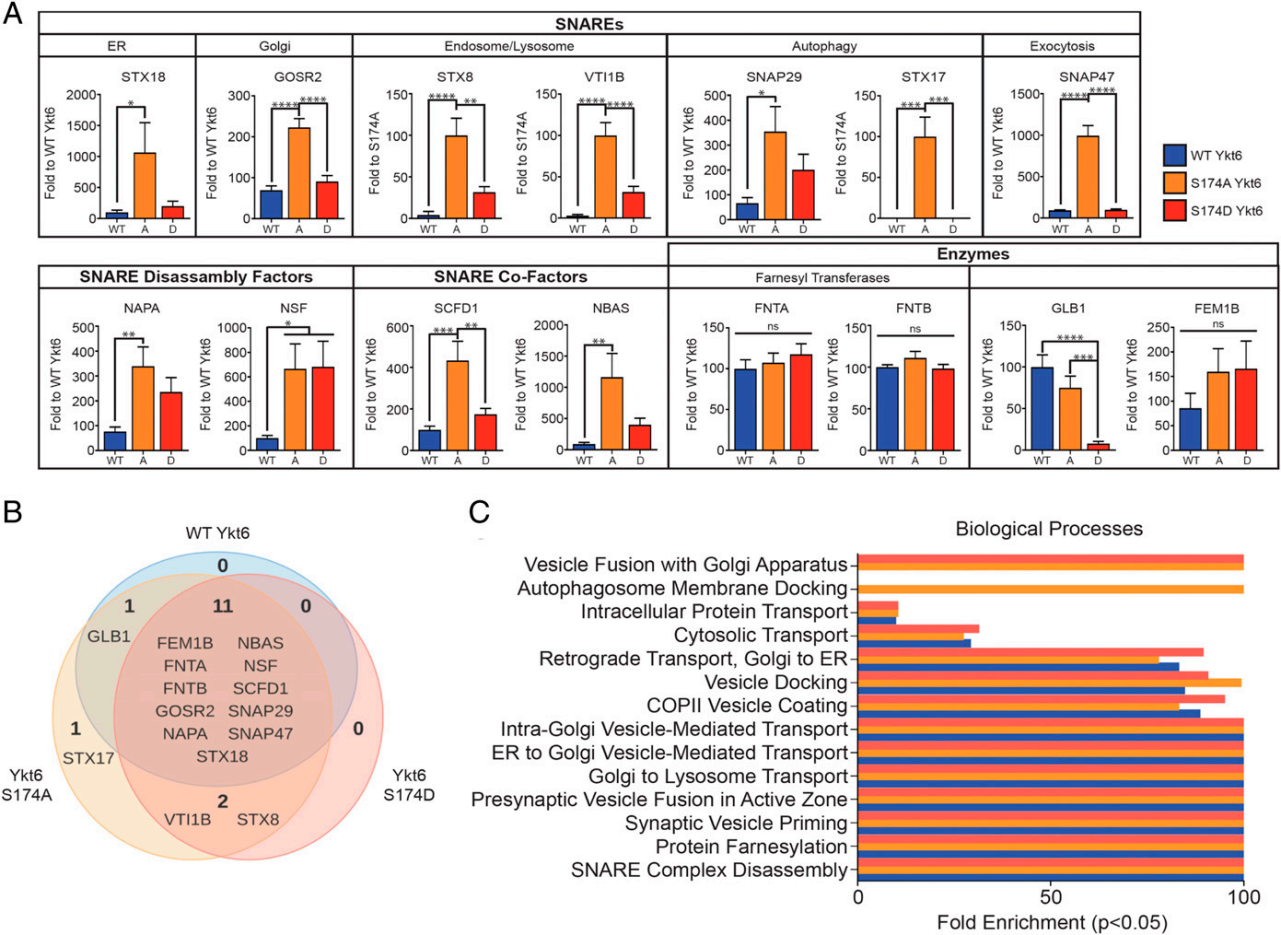
Phosphorylation of the evolutionarily conserved site in the Ykt6 SNARE domain S174 affects its specificity to its binding partners. (*A*) Quantitative analysis of MS hits that passed the selection criteria described in main text and *Materials and Methods*. Spectral counts for common interactors were normalized to WT Ykt6. *n* = 3, independent MS runs. **P < 0.01, ****P < 0.0001. One-way ANOVA, Tukey’s test. (*B*) Venn diagram representation of all MS hits that passed the selection criteria described in main text and *Materials and Methods*. (*C*) Gene ontology analysis for biological processes from the MS hits in *A* and *B*. Bar colors resemble key in *A*.

The mechanisms that regulate Ykt6 cellular activities in multiple steps of the secretory, endocytic, and autophagy-lysosomal pathways are not well understood. If phosphorylation is the first step to achieve an open conformation, we wondered if the kinetics of proteome phosphorylation regulated by CaN activity is a key signaling switch that enables Ykt6 specificity to protein interactors. To test this hypothesis, we took an MS-based approach, utilizing four different experimental conditions: 1) Ykt6 in closed conformation represented by the WT (WT equilibrium is shifted toward the closed conformation with mostly cytosolic localization and low number of phosphorylated molecules), 2) Ykt6 in open conformation with negative charge (S174D mimics both open conformation and phosphorylation), 3) Ykt6 in open conformation with no charge (S174A mimics both open conformation and dephosphorylation, i.e., CaN dephosphorylation), and 4) GFP alone to control for nonspecific protein copurification. HEK293T cells were transfected with GFP-WT human Ykt6 along with the corresponding phospho-mutants and IP using GFP. Ykt6 IP were robust and, importantly, recovered similar amounts of Ykt6 across WT and the phospho-mutants (*SI Appendix*, Fig. S5*A* and Dataset S2). We retrieved a total of 15 candidates for Ykt6-interacting partners that scored as positive hits (Fig. 4 *A* and *B* and Dataset S2).

To understand how the common hits differed between WT and phospho-mutants, we normalized the spectral counts to WT Ykt6. Between WT Ykt6 and phospho-mutants, 11 protein interactions were common; however, the ratios of these interactions changed depending on the charge (Fig. 4*A* and Dataset S2). Interactions that were dependent on Ykt6 open dephosphorylated conformation (S174A) included already-established interacting SNARE partners of Ykt6 in vesicular trafficking such as Gosr2 and Sly1 (SCFD1 gene) and in autophagy such as SNAP29 and Stx17 (Fig. 4*A* and Dataset S2). Interactions that depended on Ykt6 open conformation irrespective of the phosphorylated state (common to S174A and S174D, although higher in magnitude in S174A) included the SNAREs SNAP29 and Stx18 and cofactors α-SNAP (NAPA gene), Nbas, and the vesicle fusion ATPase NSF. Interactions that occurred irrespective of the conformational state (present in WT Ykt6, S174D, and S174A) were already-known interacting partners such as the SNARE cofactor Sly, the farnesyl transferases FntA and FntB, and newly identified ones such as the E3 ubiquitin ligase Fem1B (Fig. 4*A* and Dataset S2).

To understand how the proteins retrieved from the MS analysis reflect Ykt6 cellular activities and how these could be perturbed by the phospho-mutants, we analyzed the hits at a functional level (Fig. 4*C*). We saw enrichment in Ykt6 function in different vesicular trafficking steps such as the Golgi and ER transport as it has been described. The biological processes that were more sensitive to Ykt6 phosphorylated open conformation were autophagosome membrane docking, intra-Golgi transport, and Golgi organization (Fig. 4*C* and Dataset S2). Taken together, these MS results reveal a critical role of phosphorylation at the evolutionarily conserved site S174 in determining Ykt6 specificity to binding partners as well as cellular pathways in which phosphorylation might play a role.

### Phosphorylation at S174 Regulates Ykt6 Function in the Secretory Pathway.

**Fig. 5.**
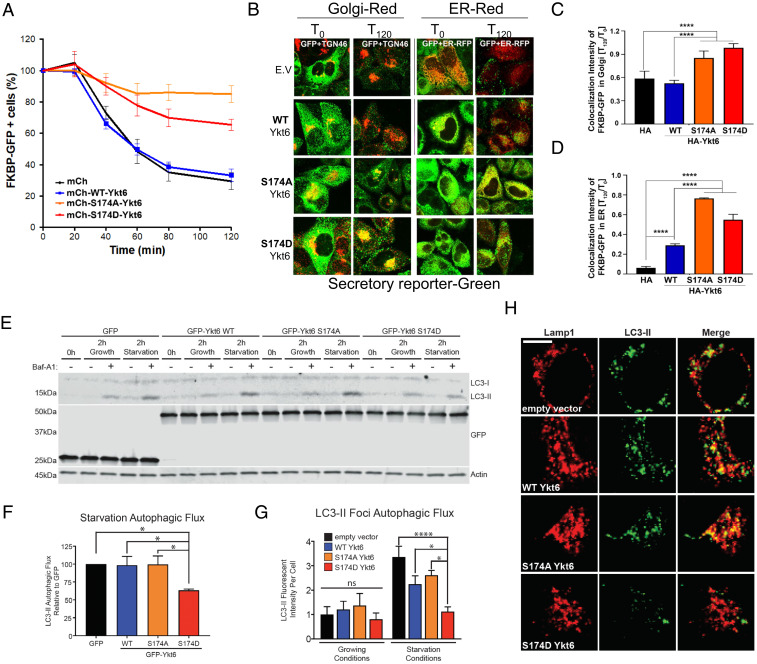
Phosphorylation of Ykt6 at the evolutionary conserved S174 plays an important role in the secretory pathway. (*A*) FACS analysis depicting the percent of FKBP-GFP–positive cells over time upon overexpression of mCherry, N-terminal mCherry-tagged of WT Ykt6, phosphoablative mutant (S174A), and phosphomimetic mutant (S174D) of YKT6 post addition of solubilizer. (*B*) Representative IF images of PC4 cells expressing FKBP-GFP reporter, transiently transfected with HA-tagged WT Ykt6 or indicated phospho-mutants of Ykt6 and immunostained with TGN-46 for *trans*-Golgi or cotransfected with mCherry-Sec61 for the ER (ER-RFP). (*C*) Colocalization analysis based on Mander’s correlation coefficient for FKBP-GFP reporter retained in *trans*-Golgi post 120 min of solubilizer in cells transfected with HA-tagged WT Ykt6 or indicated phospho-mutants of Ykt6. (*D*) Colocalization analysis based on Mander’s correlation coefficient for FKBP-GFP reporter retained in ER post 120 min of solubilizer in cells transfected with HA-tagged WT Ykt6 or indicated phospho-mutants of Ykt6. N = ∼900 cells, ****P < 0.0001. One-way ANOVA, Tukey’s test. (*E*) Representative Western blot for LC3-II from HEK293T transiently overexpressing GFP and N-terminal GFP-tagged fusion of WT Ykt6 or phospho-mutants. Cells were treated for 2 h with 200 nM Bafilomycin A_1_ (Baf-A_1_) in growing conditions (fresh 10% fetal bovine serum and 4.5% glucose growth medium) or in starvation conditions (1× Hank’s Balanced Salt Solution supplemented with 250 nM Torin-1 and 10 mM Hepes). Actin serves as a loading control. (*F*) Autophagic flux (defined as [LC3-II Baf-A_1_] – [LC3-II]) from Western blot in *A* of starvation conditions. *n* = 6; *P < 0.05, **P < 0.01, ****P < 0.0001. Two-way ANOVA, uncorrected Fisher’s LSD test. (*G*) Autophagic flux based on fluorescence intensity of GFP-LC3-II foci of overexpressed GFP-LC3 from HeLa cells expressing WT human Ykt6 and phospho-mutants, under starvation conditions with Baf-A_1_. (*H*) Representative IF images of overexpressed GFP-LC3 and endogenous Lamp-1 colocalization from HeLa cells expressing WT human Ykt6 and phospho-mutants, under starvation conditions with Baf-A_1_.

Our proteomic results showed that Ykt6 open dephosphorylated conformation (S174A) had increased binding with Gosr2 and Stx18 compared to the closed and open phosphorylated conformation (Fig. 4*A*, *SI Appendix*, Fig. S5*C*, and Dataset S2). Gosr2 normally forms a SNARE complex with Sec22, a Ykt6 genetically redundant partner (31), and facilitates ER-to-Golgi and intra-Golgi transport (32). To investigate whether the CaN-dependent phosphorylation site S174 in Ykt6 may play an active role in regulating the secretory traffic, we turned to a mammalian ligand-inducible reporter HeLa-derived cell line (33). This system utilizes a ligand-reversible dimer as a reporter of secretion consisting of a mutated FKBP protein fused to GFP, which can be quantitatively monitored using flow cytometry cell sorting (FACS). When these proteins dimerize, they form large aggregates that are retained in the ER, which results in an increase in fluorescence intensity measured. Upon the addition of an analog of rapamycin, these aggregates can be efficiently and rapidly secreted from the cells, which result in a drop in fluorescence intensity over time (*SI Appendix*, Fig. S6*A*). In order to analyze the Ykt6-positive cells, we utilized N-terminal mCherry-tagged fusion constructs of WT, S174A, or S174D human Ykt6 (*SI Appendix*, Fig. S6*B*). We saw no significant differences in the kinetics of the secretory reporter between control (mCherry alone) and WT Ykt6 over the time course (Fig. 5*A*). This is not surprising given the fact that the WT Ykt6 is primarily in an inactive, closed conformation in the cytosol. However, we saw marked differences with the phospho-mutants (Fig. 5 *A* and *B*). Both phospho-mutants showed persistently higher levels of the reporter compared to both control and WT Ykt6 over the time course, an indication of defects along the secretory pathway.

While this HeLa cell line expresses relatively low levels of endogenous Ykt6 (*SI Appendix*, Fig. S1*C*), to avoid any artifact of overexpression, we analyzed the effect of the phospho-mutants in the absence of endogenous Ykt6. To this end, we used a short hairpin RNA (shRNA) approach targeting human Ykt6 3′-UTR to knock down the endogenous Ykt6 followed by expression of human complementary DNA expressing mCherry fusions of either WT, S174A, or S174D Ykt6. Knockdown efficiency was ∼70% and did not affect the expression of exogenous Ykt6, as confirmed by the real-time qPCR analysis (*SI Appendix*, Fig. S6*C*). Reduction of endogenous Ykt6 had no significant effect in the kinetics of the secretory reporter, which is consistent with its low expression levels in this cell line (*SI Appendix*, Fig. S6 *D* and *E*). Similar to the overexpression conditions, we saw no differences between mCherry alone and WT Ykt6 over the time course; however, both phospho-mutants showed a block in the secretory pathway.

The FACS analysis, while very useful to detect overall kinetics of the secreted protein, cannot distinguish where within the secretory pathway these defects might arise, which could reflect differences between the mutants. To investigate whether these mutants might play different roles within the secretory pathway, we analyzed the presence of the reporter in both the ER and the Golgi via IF. While both phospho-mutants caused the GFP-tagged reporter to be retained in the ER and Golgi, the S174A mutant had more reporter retained in the ER, while the mutant had more reporter present in the *trans*-Golgi (Fig. 5 *B*–*D* and *SI Appendix*, Fig. S6 *F* and *G*). Together, these data suggest that phosphorylation at the S174 site in the open conformation plays a distinct regulatory role in the early steps of the secretory pathway.

### Phosphorylation at S174 Regulates Ykt6 Function in Autophagy.

Our proteomic and IP validation results showed that Stx17 and SNAP29 were enriched in the Ykt6 S174A mutant compared to the S174D mutant (Fig. 4 *A* and *B* and *SI Appendix*, Fig. S5*B*, and Dataset S2). Stx17 has been implicated in the closure of the autophagosomal membrane (34, 35), and our gene ontology analysis also pointed to a role of phosphorylation in this process (Fig. 4*C*). To investigate whether the differences in binding between Ykt6 S174A and the S174D mutant with other members of the autophagosome/lysosome fusion machinery could play a functional role in autophagy, we examined the effect of these mutants under starvation conditions. GFP-WT human Ykt6, S174A, and S174D human Ykt6 were expressed in HEK293T cells and grown in either complete media (growing conditions) or with the mTOR inhibitor Torin 1 (starvation conditions). To monitor autophagic flux, we examined the expression of the autophagy marker LC3 in the presence and absence of Bafilomycin A_1_ (Baf-A_1_), a potent inhibitor of the vacuolar H^+^ ATPase. The presence of Baf-A_1_ indicates whether there is an increase or decrease in the autophagic flux. No change in LC3-II expression or puncta indicates an inhibition in autophagy, whereas an increase in LC3-II expression or puncta indicates an increase in autophagy. By Western blot (WB), induction of autophagy increases the expression of lipidated LC3 (LC3-II), which migrates at a lower molecular weight. Under normal growth conditions, we did not observe an effect of the WT or phospho-mutants on basal levels of LC3-II or LC3-II-autophagic flux by either WB or IF (Fig. 5 *E*–*G* and *SI Appendix*, Fig. S7 *A*–*F*). However, under starvation conditions, we observed a block in autophagic flux only in the S174D mutant that was manifested by a lack of increase in LC3-II expression (Fig. 5 *E*–*G* and *SI Appendix*, Fig. S7 *G*–*I*). Furthermore, we observed very little LC3-II puncta under starvation conditions and no colocalization between LC3-II puncta and the lysosomal marker Lamp-1 in cells expressing the Ykt6 S174D mutant (Fig. 5 *G* and *H* and *SI Appendix*, Fig. S7*F*), suggesting a block in autophagosome biogenesis. To avoid possible confounding issues because of overexpression, we took a knockdown approach. Similar to the overexpression conditions, we did not observe an effect on basal levels of LC3-II or LC3-II–autophagic flux in WT Ykt6 phospho-mutants under normal growth conditions on basal levels of LC3-II or LC3-II-autophagic flux by either WB or IF (*SI Appendix*, Fig. S7 *C* and *D*). Under starvation conditions, and consistent with a nonessential role of Ykt6 in autophagy (12), we saw no defects upon knocking down Ykt6 (*SI Appendix*, Fig. S7 *C* and *D*). However, the S174D mutant blocked autophagic flux manifested by a lack of increase in LC3-II expression (*SI Appendix*, Fig. S7 *C* and *D*). Together, these data indicate that phosphorylation at the evolutionary conserved CaN-sensitive site S174 in the Ykt6 SNARE domain plays an important inhibitory role during the early steps of autophagy.

### Phosphorylation at the Yeast to Human Evolutionarily Conserved Calcineurin-dependent Site Plays a Critical Role in Models of α-Syn Toxicity.

**Fig. 6.**
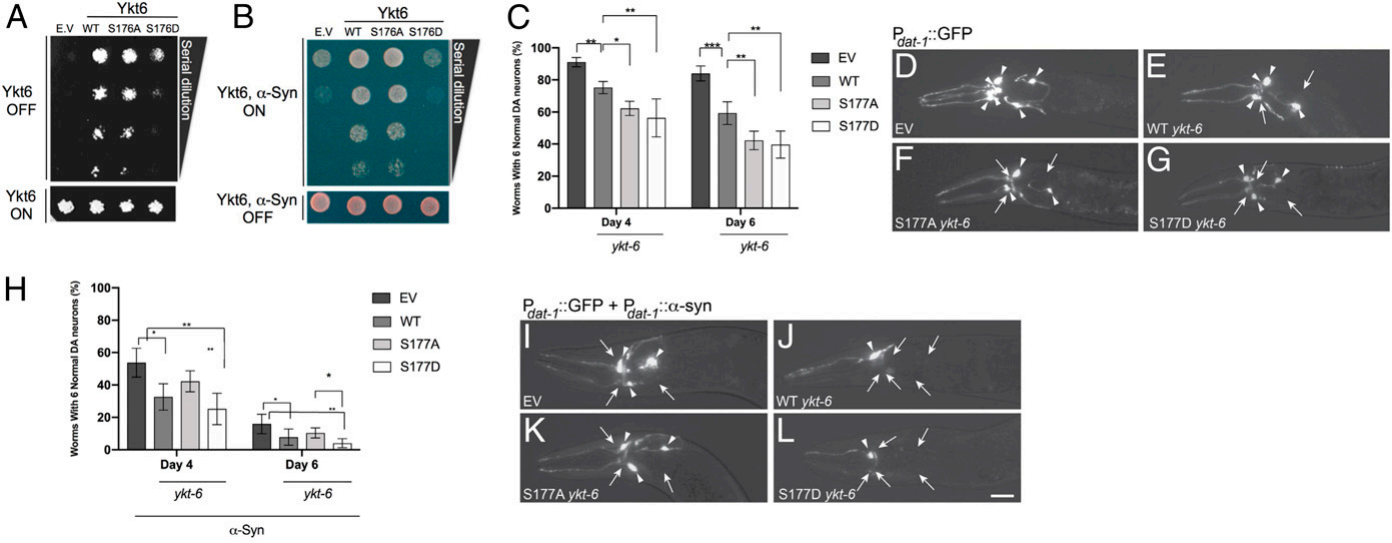
Regulation of the evolutionarily conserved CaN-dependent site is critical for cellular physiology in both yeast and *C. elegans* models of α-synuclein toxicity. (*A*) WT yeast cells were spotted onto plates containing Synthetic Defined Leu media and episomal Ykt6-Leu selective and then replica plated in fourfold serial dilutions onto episomal Ykt6-inducing plates (Galactose [SGal]-Leu; episomal-Leu selective: empty vector [EV], WT, phosphoablative [S176A], and/or phosphomimetic [S176D]). Endogenous Ykt6 is depleted by incubating the cells at 37 °C, the nonpermissive temperature. Representative plate of *n* = 3. (*B*) Yeast strains were spotted onto plates containing uninducing media (Synthetic Defined Leu; Gal-Ykt6, Gal α-syn selective; *Lower*) and replica platted in fourfold serial dilutions onto Ykt6, α-syn–inducing plates containing selective media and SGal-Leu (*Upper*). EV is used as control plasmid. Representative plate of *n* = 3. (*C*) The extent of DA neurodegeneration in *C. elegans* strains overexpressing both GFP and *ykt-6* variants (WT, S177A, and S177D) in DA neurons under the *dat-1* promoter, determined at both days 4 and 6 posthatching. Control worms overexpressed both GFP and the EV backbone containing the *dat-1* promoter, with *ykt-6* variants omitted. Data are shown as the average of three independent stable transgenic lines for each construct, with each line in triplicate. Error bars indicate SD. One-way ANOVA with a Tukey’s post hoc test. *P < 0.05, **P < 0.01, ***P < 0.001. (*D*–*G*) Representative images of *C. elegans* neurons taken at day 6 posthatching in the backgrounds described in *D*. The anterior (head) portion of worms are shown, allowing the visualization of the six DA neurons in this region via GFP fluorescence. Arrowheads indicate intact DA neurons. Arrows indicate degenerated DA neurons in a strain overexpressing control vector and GFP (*D*), WT *ykt-6* and GFP (*E*), phosphoablative S177A *ykt-6* and GFP (*F*), or phosphomimetic S177D *ykt-6* and GFP (*G*). (*H*) same as in *C* but in *C. elegans* strains overexpressing GFP, α-syn, and *ykt-6* variants (WT, S177A, and S177D) in DA neurons under the *dat-1* promoter, determined at both days 4 and 6 posthatching. (*I*–*L*) same as in *D*–*G* in a background overexpressing GFP, α-syn, and *ykt-6* variants (WT, S177A, and S177D) in a strain overexpressing EV control vector, GFP, and α-syn in the DA neurons where three neurons are missing (*I*); WT *ykt-6*, GFP, and α-syn where five DA neurons are missing (*J*); phosphoablative S177A *ykt-6*, GFP, and α-syn in the DA neurons where three neurons are missing (*K*); or phosphomimetic S177D *ykt-6*, GFP, and α-syn in the DA neurons where five dopaminergic neurons are missing (*L*).

The basic mechanisms of the secretory pathway are well conserved between yeast and mammals (36, 37). Therefore, we next asked if modulating the phosphorylation of the yeast to human evolutionarily conserved phosphosite could balance toxic versus protective responses to α-syn in yeast. Our functional data from mammalian cells indicated that both phospho-mutants caused defects along the secretory pathway, albeit in different steps. Since yeast are highly reliant on the secretory pathway for living, if the evolutionarily conserved phosphosite we identified plays an important role in Ykt6 function in yeast, then both phospho-mutants should decrease yeast viability relative to WT Ykt6. To test Ykt6 physiological relevance, we took a genetic approach and generated phospho-mutants for the yeast equivalent evolutionarily conserved site S176. Yeast WT Ykt6, S176D, and S176A mutants were expressed under a galactose-inducible promoter. As expected, overexpression of the yeast S176D mutant profoundly compromised the ability of the yeast cells to grow (*SI Appendix*, Fig. S8*A*). To our surprise, however, the yeast mutant S176A did not. In fact, Ykt6 S176A had a marginal increase in cell growth compared to WT Ykt6 (*SI Appendix*, Fig. S8*A*). To avoid confounding issues because of overexpression, we carried out the same experiments in the absence of endogenous Ykt6. Since Ykt6 is an essential gene, we used a yeast temperature-sensitive strain whereby expression of Ykt6 is abolished at a nonpermissive temperature (37 °C) leading to growth defects (38). Episomal expression of WT Ykt6, but not the control plasmid, rescued the growth defects caused by the lack of endogenous Ykt6 (Fig. 6*A*). Expression of the S176D mutant did not complement cell growth. Expression of the S176A, however, was still able to rescue viability to the extent of the WT (Fig. 6*A*).

Next, we asked if modulating the phosphorylation of yeast S176 could balance toxic versus protective responses to α-syn. Inducing the expression of human α-syn in yeast cells leads to cellular responses relevant to the α-syn–mediated pathologies observed in neurons. These include defects in vesicle trafficking (28, 39), defects in autophagy (40, 41), and Ca^2+^-CaN dysregulation, among others (25, 27). It was previously shown that overexpression of Ykt6 and/or other genes involved in ER–Golgi forward vesicular trafficking could rescue α-syn toxicity in yeast (28). If the S176A mutant promotes anterograde vesicular trafficking from the ER, we expected that overexpression of WT Ykt6 and S176A mutant would rescue α-syn toxicity, whereas the Ykt6 S176D mutant would not. Indeed, overexpression of WT Ykt6 and S176A mutant rescued α-syn toxicity, whereas overexpression of the S174D enhanced α-syn toxicity (Fig. 6*B*).

We next asked if WT Ykt6 or phospho-mutant variants had any effect in dopaminergic (DA) neurons, the cell type most classically implicated in PD. Because Ykt6 is also essential in *C. elegans*, to first assess the effects of Ykt6 and its phospho-mutants in DA neurons in vivo, we overexpressed GFP and the *C. elegans* homolog of Ykt6 (*ykt-6*) under the transcriptional control of the DA neuron-specific dat-1 promoter. *C. elegans* hermaphrodites invariably have six DA neurons in the anterior region of the animal that do not display significant neurodegeneration over time (Fig. 6*D*). If *C. elegans* DA neurons are as reliant on the secretory pathway as mammalian cells, we expected to see less DA neurons in the presence of either phospho-mutant compared to the WT *ykt-6*. Three separate transgenic lines were created and analyzed for each variant. Indeed, overexpression of both *C. elegans ykt-6* phosphoablative or phosphomimetic mutants of the evolutionarily CaN-sensitive site (S177) caused some neurodegeneration compared to the WT (Fig. 6 *C*–*G*). However, these neurons were also very sensitive to *ykt-6* dosage since overexpression of WT *ykt-6* alone was sufficient to cause a mild neurodegeneration (Fig. 6 *C*–*E*). We observed a similar trend in neurodegeneration at both day 4 and day 6 posthatching, with the severity increasing overall by day 6, indicating the increasingly neurotoxic nature of these *ykt-6* variants (Fig. 6*C*). To assess the effects of these mutants in the presence of α-syn in vivo*,* we took advantage of a previously established nematode model of α-syn toxicity (42). In this case, transgenic *C. elegans* expressed GFP, *ykt-6*, and α-syn under the transcriptional control of the DA neuron-specific dat-1 promoter. Three separate transgenic lines were created and analyzed for each variant. As previously reported, expression of α-syn caused an age- and dose-dependent degeneration of DA neurons (28) (Fig. 6 *H* and *I*). Since WT *ykt-6* was already toxic to normal DA neurons, as expected, its overexpression in the presence of α-syn enhanced toxicity (Fig. 6 *H*–*J*). Similarly, overexpression of the *C. elegans* S177D furthered enhanced α-syn toxicity relative to WT *ykt-6* (Fig. 6 *H* and *L*). We observed a similar trend in neurodegeneration at both day 4 and day 6 posthatching, with the severity increasing overall by day 6, indicating both the increasingly neurotoxic nature of these *ykt-6* variants and α-syn expression (Fig. 6*H*). Surprisingly, the S177A mutant, while it did not protect DA neurons against the toxic effects of α-syn as it did in the yeast cells, was less toxic than the WT *ykt-6* (Fig. 6 *H*–*K*).

These collective in vivo results from yeast and *C. elegans* show how the regulation of Ykt6 through phosphorylation/dephosphorylation at the evolutionary conserved CaN-dependent phosphosite impacts normal cellular physiology, highlighting evolutionary differences. Additionally, the functional consequences of Ykt6 modulation under cytotoxic conditions induced by transgenic overexpression of the PD-associated gene product, α-syn, further signifies the importance of Ykt6 as a mediator of neurodegenerative pathogenesis, such as PD.

## Discussion

Through a multidisciplinary approach encompassing both in vitro and in vivo analyses in yeast, worms, and human cells, we reveal a regulatory mechanism by which Ykt6 operates. Specifically, we demonstrate that phosphorylation at an evolutionarily conserved site within the Ykt6 SNARE domain is a key regulatory step that drives a conformational change from a closed cytosolic form to an open membrane-bound form. Our structural bioinformatics studies showed that the mechanism of conformational change involves a buildup in electrostatic potential because of the synergy of phosphorylation at the yeast–animal evolutionarily conserved site and the conserved negative patch located in the same structural vicinity. The electrostatic repulsion between the longin and SNARE domains ultimately leads to the open conformation (*SI Appendix*, Fig. S8*B*, step one). Phosphorylation of the mammalian conserved phosphosites S172 and T179 could be the first step to open Ykt6, followed by phosphorylation of the yeast–human evolutionarily conserved site S174, which may play a key role in stabilizing the open conformation and allow Ykt6 to insert into a membrane (*SI Appendix*, Fig. S8*B*, step two). Supporting our findings, a phosphorylation driven membrane recruitment for Ykt6 was also reported while this paper was under review (43). The conformational mechanism of activation might not be restricted to Ykt6 since the S174 phosphorylation site is also evolutionarily conserved across other R-SNARE proteins (44). This suggests that the conformational change caused by phosphorylation within the SNARE domain might be a general regulatory mechanism of other R-SNAREs and prompts further investigation.

We also demonstrated that the conserved phosphorylation within the Ykt6 SNARE domain is regulated by Ca^2+^ signaling through kinases like PRKCi and the phosphatase CaN. A pharmacological approach to modulate their activity would have been difficult since both enzymes have many substrates, and this approach will not detect only Ykt6 phospho-specific effects. Thus, the most unambiguous approach is to individually modify the phosphosites by generating phospho-mutants to unequivocally establish the contribution for each site. The Ykt6 S174D mutant enabled us to uniquely dissect the contribution of the charge at the S174 position in the open conformation. The Ykt6 S174A mutant, while able to open up because of structural constraints posed by the hydrogen bonding naturally formed by the serine in the SNARE domain with its counterpart in the longin domain (Fig. 2*E*), could not be phosphorylated due to the presence of the alanine. Using these mutants, we found that phosphorylation perturbs the hydrophobic interactions at this layer. Reducing SNARE interactions and/or interfering with further SNARE zippering/membrane fusion provides selectivity among Ykt6 interacting partners (*SI Appendix*, Fig. S8*B*, step three). The S174A mutant had an increased number of interactions with SNAREs and SNARE cofactors (Fig. 4). This is not surprising given that most SNAREs rely on a hydrophobic core for SNARE complex formation and would explain why the S174D mutant, which introduces a negative charge, showed less SNARE interactions when compared to the S174A mutant. Importantly, while we retrieved known Ykt6 binding partners such as Gosr2, we also retrieved new binding partners such as SNAP47, among others, which would indicate novel roles for Ykt6 in other cellular pathways such as exocytosis, prompting further investigation. These data suggest that Ykt6 specificity to its interactors might depend on the kinetics of phosphorylation/dephosphorylation and ultimately dictate the membrane compartment in which Ykt6 is active.

We showed functional consequences of the role of phosphorylation in Ykt6 open conformation in two distinct scenarios: autophagy and the secretory pathway. Locking Ykt6 in a permanent phosphorylated or dephosphorylated open conformation had aberrant consequences in the early steps of the secretory pathway. This could be explained by changes in the stoichiometry of SNARE complexes in the anterograde and retrograde traffic between the ER and Golgi. While the open conformation on the dephosphorylated state (S174A) showed the highest affinity to Gosr2 and Stx18, the open conformation phosphorylated state (S174D) failed to bind them. This, however, yielded different effects in survival outcome in yeast versus DA neurons in *C. elegans*. While survival of *C. elegans* DA neurons was compromised in both phospho-mutants relative to the WT, only the open conformation on the phosphorylated state compromised survival in yeast. Why is the yeast Ykt6 in the open dephosphorylated conformation behaving more like WT and different from neurons? One plausible explanation for this discrepancy could be the presence of the ERGIC pathway, which is not present in yeast and where Ykt6 has been shown to play a role (45). If the phosphoablative mutant engages in the retrograde traffic from the Golgi to ERGIC as suggested by its strong interaction with Stx18, it might prevent anterograde traffic only in neurons and mammalian cells but not in yeast. Furthermore, given the postmitotic nature of neurons, this may increase their sensitivity to any decreases in fitness compared to yeast. Interestingly, PRKCi, which we identified as a kinase hit for Ykt6, has been proposed to have roles in the ERGIC pathway. Future work would need to be performed to understand the role of Ykt6 phosphorylation in the ERGIC anterograde and retrograde traffic.

We revealed that Ykt6 conformational change by phosphorylation is also an important regulatory step that mediates the of critical members of the autophagosome machinery to complete starvation-induced autophagy. Specifically, we found that the Ykt6 S174D mutant was unable to bind Stx17 and played an inhibitory role in autophagosome formation indicated by the lack of LC3-II positive puncta and decreased autophagic flux (Fig. 5 *E*–*H* and *SI Appendix*, Fig. S7). Stx17 has been shown to play a role during autophagy initiation by assembling protein complexes necessary for the preautophagosomal structure (mPAS) (46). Moreover, it was recently shown that in yeast, phosphorylation of Ykt6 by Atg1 kinase also plays an inhibitory role during autophagosome formation, and dephosphorylation of Ykt6, by an unknown phosphatase, is necessary to complete autophagosome formation (47). Our data fit with these observations and suggest a model in which the Ca^2+^-CaN cascade posttranslationally regulates the early phases of autophagy through regulation of Ykt6 activity. Ykt6 phosphorylation would trigger the open conformation and its recruitment to ER-derived membranes. Subsequent dephosphorylation by CaN would enable Ykt6 interaction with Stx17 to form the mPAS and ultimately autophagosomes.

Defects in Ca^2+^ homeostasis, the secretory pathway, and autophagy have been implicated in PD (25–27, 48). Defects in Ykt6 function have also been linked to the pathobiology of α-syn (28, 30). We found that Ykt6 regulation by CaN has critical consequences in two Ykt6 activities that are deeply rooted in α-syn pathobiology: autophagy and the secretory pathway. While there may be differences in the reliance on these vesicular pathways across evolution, in the context of α-syn, our in vivo studies in *C. elegans* model pointed to the fact that DA neurons are very sensitive to both Ykt6 dosage and the phosphorylated state of the evolutionarily conserved phosphosite. Specifically, “locking” Ykt6 in either side of the spectrum (constitutive dephosphorylated state with the phosphoablative mutant or constitutive phosphorylated state with the phosphomimetic mutant) has deleterious consequences, albeit to a different extent between phospho-mutants, especially in the context of α-syn. Our functional studies indicate that shifting the equilibrium to either of these states yields profound impairments in the secretory pathway and in autophagy. Whether phosphorylation of Ykt6 affects other pathways elucidated through our MS analyses with effects on neuronal health remains to be clear. Our studies on Ykt6 regulation highlight the importance of achieving the right equilibrium in the Ykt6 phosphorylation state to achieve proper function. We had previously shown that tuning down CaN activity pharmacologically with low doses of tacrolimus could rebalance CaN toxic activity toward a protective response (25, 27). Ykt6 could be one of the key substrates that reflect CaN activity. Too much CaN activity shifts the equilibrium of Ykt6 toward the dephosphorylated form, which is toxic. Complete inhibition of CaN shifts the equilibrium of Ykt6 toward the phosphorylated form, which is also toxic. However, partial inhibition of CaN would maintain Ykt6 equilibrium between a phosphorylated and dephosphorylated state and subsequently provide neuroprotection in the context of α-syn overload. In defining the structure–function relationships underlying distinctions in Ytk6 activity, our studies provide insight into key aspects of PD pathology and inform translational efforts toward a therapeutic route for the disease.

## Materials and Methods

### Primary Cells and Strains.

Yeast strains containing α-syn were generated and induced as previously described (28). Yeast strains containing Ykt6 T^S^ allele were generated as previously described (38). Secretory reporter PC4 cell line was generated as previously described^35.^ *C. elegans* lines were generated as previously described (42). For detailed description, see *SI Appendix, Materials and Methods*.

### MS.

Relative phosphopeptide quantification by label-free shotgun proteomics was performed in collaboration with Paola Picotti at the Swiss Federal Institute of Technology in Zurich as previously described (27). iTRAQ MS was performed by the Massachusetts Institute of Technology Koch core facility. Tandem MS was carried out in Jeffrey Savas’s laboratory at Northwestern University as previously described (49). For detailed description see *SI Appendix, Materials and Methods*.

### Protein Purification and NMR.

Ykt6 6XHis-tagged variants were expressed in *Escherichia coli* construction of 6XHis-tagged YKT6 variants. For chemical shift assignments, freshly prepared samples ^13^C/^15^N-labeled Ytk6 were exchanged into a solution. All NMR spectra on the ^13^C/^15^N-labeled Ytk6 sample bound to DPC were acquired on Bruker Avance III or Varian VNMRS spectrometers operating at 900 and 600 MHz (1H). For detailed description, see *SI Appendix, Materials and Methods*.

### Molecular Biology.

Ykt6 mutants were made by Q5 site-directed mutagenesis, and shRNA against YKT6 human 3′-UTR was performed as described in detail in *SI Appendix, Materials and Methods*.

### Cellular Assays.

The secretory, autophagy, and Ca^2+^ induced assays were performed as described in *SI Appendix, Materials and Methods*.

## Supplementary Material

Supplementary File

Supplementary File

Supplementary File

## Data Availability

All study data are included in the article and/or supporting information.
