# Supplementary material for: A conformational switch driven by phosphorylation regulates the activity of the evolutionarily conserved SNARE Ykt6
Source: Proc Natl Acad Sci U S A. 2021 Mar 15;118(12):e2016730118. doi: 10.1073/pnas.2016730118 (PMC8000380; doi:10.1073/pnas.2016730118)
Supplement: Supplementary File [file pnas.2016730118.sapp.pdf]

PNAS
www.pnas.org

Supplementary Information for

"A conformational switch driven by phosphorylation regulates the activity of the evolutionarily conserved SNARE Ykt6"

Kaitlyn McGrath[1+], Shivani Agarwal[1+], Marco Tonelli[2], Mykola Dergai[3], Anthony L. Gaeta[4], Andrew Shum[1], Jessica Lacoste[5], Yongbo Zhang[6], Wenyu Wen[7], Daayun Chung[1], Grant Wiersum[8], Aishwarya Shevade[9], Sofia Zaichick[1,10], Damian B. van Rossum[11,12], Ludmilla Shuvalova[8], Jeffrey N. Savas[1], Sergei Kuchin[9], Mikko Taipale[5,13], Kim A. Caldwell[14], Guy A. Caldwell[14], Dirk Fasshauer[3,15], Gabriela Caraveo[1]*.

Gabriela Caraveo, corresponding author
Email: gabriela.piso@northwestern.edu

This PDF file includes:

Materials and Methods
Supplementary text
Figures S1 to S8
Legends for Datasets S1 to S2
SI References

Other supplementary materials for this manuscript include the following:

Datasets S1 to S2

MATERIALS AND METHODS

Cells line and manipulations

All cell lines were cultured at 37°C and 5% CO^2. HEK293T (human embryonic kidney cells) and HeLa (human cervical cancer cells) cells were cultured in Dulbecco's Modified Eagle Medium with 4.5% glucose (DMEM; Corning), 10% Fetal Bovine Serum (FBS; Denville) and 1X penicillin and streptomycin (Gibco). Hela secretory

line, PC4 cells [1] were cultured in DMEM, 10% FBS and puromycin (1 μg/mL, Sigma Aldrich). CMV-GFP-LC3 stable HeLa cell line was cultured as described above with G418 (0.1 mg/ml, Roche). PC12 (rat pheochromocytoma) cell line was stably infected with a lentivirus carrying a doxycycline inducible rat Ykt6 shRNA (targeting sequence: CAGTCGAAAGCCTTCTATA, catalogue number V3SR11254-241803912 from Dharmacon) and cultured in DMEM, 1% FBS, 10% Nu-serum (Corning), 1X penicillin and streptomycin, and puromycin (1 μg/mL, Sigma Aldrich).

Transfections were performed using either Jetprime (VWR; 89129-924) or Lipofectimine3000 (Thermofisher; L3000015) according to the manufacture's protocol. Cells were seeded at 55K cells/well in a 24-well plate, and 300K cells/well in a 6-well plate. Autophagy was induced by treating the cells for 2 hours in 250nM Torin-1 (R&D Systems, 4247/10) in 1X Hanks' Balanced salt solution, HBSS, with Calcium and Magnesium (Corning, 21-020-CV) supplemented with 10 mM HEPES buffer (Corning, 25-060-CI) at 37°C with 5% CO^2. To block lysosomal degradation cells were treated with 200 nM Bafilomycin A_1 (Baf-A_1, Cayman Chemical, 11038) for 2 hours. For all calcineurin related experiments, cells were treated for 30 minutes with 1 μM of Ca^{2+} ionophore ionomycin (Sigma, 407952-1MG) and/or 1 μM of calcineurin-specific inhibitor Tacrolimus (Ontario Chemicals Inc., 104987-11-3).

Virus Packaging

HEK293T cells were transiently transfected as described above with three constructs: pMD and pax2 (both required for viral packaging), as well as the desired lentiviral construct being packaged. Transfected cells were incubated at 37°C and 5% CO^2 for 48 hours and supernatants were collected and spin-cleared from cell debris at 500g for 10 min.

Spin Infection

PC12 cell media was exchanged for packaged virus-containing media supplemented with 0.8 μg/mL polybrene. PC12 cells were subsequently spun at 2250RPM for 2 hours at room temperature. After spin, virus-containing

media was discarded and replaced with PC12 full-growth media. PC12 cells were assayed 48 hours post infection.

PC12 rat Ykt6 shRNA Stable Cell Line

PC12 cells were infected as described above (spin Infection) and 48 hours post-infection treated with puromycin (1 µg/mL) for selection of the ShRNA.

Ykt6 Knock Down

HeLa PC4 cell line expressing mutated FKBP-GFP reporter was co-infected with viruses packed using either ShRNA against YKT6 (Sigma YKT6 MISSION shRNA (SHCLNG-NM_006555TRCN0000380048), human 3'-UTR (GTACCGGTGGGCAAATGAAACCATAAACCTCGAGGTTTATGGTTTCATTTGCCCATTTTTT), clone ID (NM_006555.3-985s21c1) or scrambled ShRNA (scramble shRNA was a gift from David Sabatini (Addgene plasmid # 1864 ; http://n2t.net/addgene:1864 ; RRID:Addgene_1864) [2] sequence as control along with GFP-tagged human Ykt6 and phospho-mutant constructs. The method used for Virus Packaging and Spin Infection is described above.

Ykt6 shRNA stable PC12 cell line was infected with GFP-tagged human Ykt6 and mutants as described in Virus Packaging and Spin Infection. After 2-hour spin infection, virus-containing media was discarded and replaced with PC12 full-growth media supplemented with 10ng/mL doxycycline for 48 hours to allow the induction of the shRNA.

PC4 Secretory assay

PC4 cells [1] were incubated with 0.5 µM D/D Solubilizer (Takara; 635054) in a time course-dependent manner. D/D Solubilizer was deactivated by incubating the cells for 10 minutes on ice and subsequently trypsinized in 0.25% EDTA (Corning) on ice for an additional 30 minutes. All supernatants were collected and analyzed by fluorescence activated cell sorting (FACS) for both, mcherry (Ykt6 transfected) and GFP (secretory reporter) positive cells.

Real-time polymerase chain reaction (RT-PCR/Q-PCR)

RNA was extracted from cells using EZNA total RNA kit 1 (Omega BioTrek, Norcross, GA). Random hexamers, M-MLV reverse transcriptase (Thermo Fisher Scientific) and 1 µg of total RNA were used for reverse transcription. Primers to detect overexpressed YKT6 (Forward-5'-CAGCGTCCTCTACAAAGGCG-3' and Reverse-5'-ACAATCAGTTGACTCGTGAAGG-3') and the 3'-UTR primer to detect endogenous YKT6 (Forward-5'- CATCTGAACAAGTGGGTCTCTC-3' and Reverse-5'- TGGGCAGACTGGAACATAAC-3') and the GAPDH (Forward-5'- GTCTCCTCTGACTTCAACAGCG-3' and Reverse-5'- ACCACCCTGTTGCTGTAGCCAA-3') as the housekeeping gene were used. Q-PCR was performed with SsoAdvanced™ Universal SYBR Green Supermix (Bio-Rad Laboratories, Hercules, CA). The real-time RT-PCR reactions were carried out using Roche LightCycler® 480 system. Fold change was calculated as 2-ΔΔCt and the values were normalized to the housekeeping GAPDH transcript.

Subcellular fractionation

Subcellular fractionation was carried out on transfected HEK cells collected from 95% confluent 10cm dishes. Briefly, collections were pelleted, washed with cold 1xPBS, and lysed via probe sonicator (8 second pulse) two times in lysis buffer containing sucrose and HEPES. Cytosolic and membrane fractions are separated via ultracentrifugation at 10,000 x *g* at 4°C for 10 min. After washing and resuspending pellets, 15 micrograms of the total cell input, membrane and cytoplasmic fractions were loaded on SDS-PAGE and transferred and probed with mouse anti-GFP antibody (Santacruz, 1:2000). Blots were further probed with rabbit anti-Na+/K+ ATPase α-3 antibody (Millipore Sigma, 1:1000) and rabbit anti-tubulin (Cell signaling, 1:10,000) to assess the purity of the membrane and cytoplasmic fractions, respectively. IRDye 800CW goat anti-rabbit 1018 and IRDye 680 goat anti-mouse were used as secondary antibodies (Li-COR, 1:10,000). Blots were developed using Odyssey CLx imaging system (LI-COR Biosciences). Analysis consisted of integrated densitometry of appropriate bands. YKT6 density values were normalized to respective loading controls (Na+/K+ ATPase α-3 for membrane fractions and tubulin for cytosolic fractions). Ratios were calculated using normalized membrane/cytosolic fractions.

Analysis for membrane localization

HeLa cells were seeded on 18mm poly-D-lysine coated coverslips and co-transfected with either YKT6 WT or YKT6 S174D and 1 of 4 FLAG-tagged candidate kinases (PRKCi, SGK, CAMK1G, CAMK2B). After pharmacological treatment with DMSO or 1uM ionomycin + 1uM FK506, cells were fixed in cold 4% PFA in 1X PBS. Coverslips were stained for anti-GFP (Santacruz, 1:1000) and anti-FLAG (Sigma, 1:1000). Appropriate fluorescent secondaries were applied and mounted for imaging using confocal microscopy. Z-stack images were acquired and subsequently processed using a custom script. Line scan analysis over 6 points on Feret's diameter of isolated cells are accumulated and the local maxima and minimum were calculated. Ratios based on these numbers operate as proxies for membrane/cytosol intensity measurements allowing for whole cell evaluation of membrane changes in YKT6. Concurrently, confocal images are evaluated by technicians under two independent blinded analyses. Membrane and cytosolic values were incorporated into the final statistical analysis (t-Test, ANOVA, etc).

High-Content Screen

Cell culture

HeLa Kyoto cells (female) used in this study were a gift from the Cheeseman laboratory (Whitehead Institute, Cambridge, MA) and cultured in DMEM with 10% FBS and 1% penicillin/streptomycin. Cells were dissociated with trypsin and all cells were maintained at 37 °C and 5% CO_2. Cells were regularly monitored for mycoplasma infection. HeLa Kyoto cells were authenticated with STR profiling (GenePrint 24 System, Promega) at The Centre for Applied Genomics, The Hospital for Sick Children, Toronto.

Plasmids

We used the human kinase collection previously reported [3]. Briefly, all kinases were cloned into a mammalian expression pCDNA3.1 plasmid with a C-terminal FLAG and V5 tags (Addgene plasmid #87063).

Transfection and Immunofluorescence

Cells were seeded on CellCarrier-96 well black, optically clear bottom plates (Perkin Elmer) at a density of 5,000 cells/well and incubated overnight to attach. Plasmids were co-transfected into HeLa Kyoto cells with

XtremeGENE 9™ (Roche) following the manufacturer's protocol. Two days post-transfection, cells were fixed with 4% paraformaldehyde in culture medium for 20 min at room temperature. Cells were permeabilized with 0.1% Triton X-100/1xPBS for 10 min and blocked with 1% BSA/0.1%Triton X-100/1x PBS for 1 hour. Cells were incubated with anti-FLAG M2 antibody (1:500, Sigma-Aldrich) for 1 h at room temperature. Subsequently, cells were incubated with Alexa Fluor 647 goat anti-mouse (1:500, Thermo Fisher Scientific), Hoechst 33342 (1:5000, Thermo Fisher Scientific), and Wheat Germ Agglutinin, Alexa Fluor™ 594 Conjugate (1:500, Thermo Fisher Scientific) for 1 hour.

Image acquisition and analysis

For quantitative imaging of stained cells, images were acquired using the Opera Phenix™ screening system (PerkinElmer) with a 63× water objective. In every experiment twenty-five fields were acquired per well with Harmony™ high-content imaging software (PerkinElmer). Further analysis was done with the Harmony™ analysis system and Columbus™ data storage system (PerkinElmer). Basic flatfield correction and maximum projection of images was performed. Nuclei were identified from the Hoechst stained channel (Method B) and cell boundaries were defined based on GFP staining of YKT6 and the nuclei (Method F). Mean intensities of cells were calculated for the 488 channel and the 647 channel, and only cells with an average intensity of >250 in 488 and >200 in 647 were selected for further analysis. Cells on the borders of the field-of-view were removed. Intensity was measured in the whole cell, area of the cytoplasm, area of the membrane, and a 55% boundary surrounding the outside of the nucleus.

Semi-automated kinase screen image analysis

Images were background subtracted and processed for ImageJ scripted intensity analysis. Briefly, nuclei were used to define cells and direct voronoi segmentation. Cells that had at least 50% intensity above the background were selected for further processing. Wheat Germ Agglutinin (WGA) signal thresholder by 90% was used as the mask to define the ROIs. Ykt6-GFP signal was summed up over the mask per cell. Integrated intensity values were averaged over entire fields and processed for the 300 kinase clones. Results are displayed as fold-change over YKT-6 WT values.

Colocalization analysis

Confocal images were processed using ImageJ. Images were background subtracted and processed for Voronoi decomposition using DAPI as a reference (for proper separation of cells) These processed images were analyzed using an automated cell counting program with the following parameters: 1.) cells with an FKBP12 threshold of 100% above background, 2.) cells containing positive signal for Ykt6 threshold of 75% above the background, 3.) cells that contain DAPI with a circularity index between 0.7-1 (healthy, non-dividing nucleus), 4.) exclusion of cells with total cell localization of either Golgi or ER markers or a circularity index of 0.92-1 (indicating a dead or dying cell). Pixel intensity of secretory reporter FKBP12 was measured using either the Golgi or ER signal as a mask and consolidated over the Z stack of the cell. Cell counts and values were validated independently by manual cell count of one stack per condition and duplicates were removed by excluding those with identical bounding box parameters (indicating overlapping X-Y cell coordinates between counts). Cells counts and intensity values were exported for statistical analysis. Ratios were calculated using values for T120 over T0.

Yeast maintenance and manipulations

We utilized two types of yeast strains: W303 (for the overexpression experiments) and BY4742 Ykt6 Temperature sensitive strain (Ts) [4]. To transform the Ykt6 constructs into both strains, yeast cells were grown overnight to saturation at 30°C in a shaking incubator. The following day, the culture is diluted to 0.1 OD_{600}. When cultures reach 0.4-0.6 OD_{600}, they were centrifuged at 4,000g and washed with autoclaved deionized water. The final pellet was resuspended in 0.1 M lithium acetate (LiAc) and incubated at 30°C for 15 minutes, followed by the addition of the plasmid DNA, salmon sperm DNA mixture along with a 50% polyethylene glycol (PEG) solution (4 mL 50% PEG; 0.5 mL 1 M LiAc; 0.5 mL water). Cells+DNA were incubated at 30°C for 30 minutes, 42°C for 22 minutes, then centrifuged and washed of any remaining PEG solution. Transformants were spread onto plates with synthetic dropout (SD) medium containing glucose and lacking relevant amino acids for selection. Plates were incubated at 30°C for 3 days. For spotting assays, yeast cell cultures were grown overnight at 30°C in SD medium lacking the relevant amino acids and containing glucose. Cell concentrations

(OD_{600}) were adjusted to the concentration 0.05 OD_{600}, five-fold serially diluted, and spotted onto SD medium plates containing either glucose (uninduced) or galactose (induced). For the overexpression experiments, plates were incubated at 30°C and 37°C (for the Temperature sensitive strain) for 2 days (glucose) or 3 days (galactose).

Plasmids and primers

ER-RFP was purchased from Addgene (62236). Yeast Ykt6 (Dharmacon, YSC4613), was mutated using Q5 Site-Directed Mutagenesis Kit (NEB; E0554S). Yeast Ykt6 was mutated using the following primers: Forward 5'-GGTGGACAAAGCGGAGTCATT-3' and Reverse 5'-AAATTATCCAACTTTTCACCTCTTTG-3' sequences to generate the S176>A substitution and Forward 5'-GATGAGTCATTAACGGCAAGTTC-3' and Reverse 5'-ATCTTTGTCCACCAAATTATC-3' sequences to generate the S176>DD substitution. Human eGFP-Ykt6 (a kind gift from Dr. Joseph Mazzulli, Northwestern University), was mutated using Q5 Site-Directed Mutagenesis Kit with the following primers: Forward 5'-GGTGTCCAAAGCCGAGGTGCTGG-3' and Reverse 5'-AAGTCATCTAGCTTCTCACCTCGC-3' sequences to generate the S174>A substitution, Forward 5'-GGTGTCCAAAGACGAGGTGCTGG-3' and Reverse 5'-AAGTCATCTAGCTTCTCAC-3' sequences to generate the S174>D substitution, Forward 5'-AAAACTCATGtccTGTGCCATCATG-3', and Reverse 5'-GTTTCCGGGCAGTTTTATAG-3' to generate C194S, Forward 5'-ACTCATGCTGtccGCCATCATGATG-3'; and Reverse 5'- TTTGTTTCCGGGCAGTTT-3' to generate C195S and Forward 5'-AAACTCAAGCagtGCCATCATGATG-3' and Reverse 5'-TGTTTCCGGGCAGTTTTATAG-3' to generate C194S_C195S double mutant. mCherry-Ykt6 constructs were made by subcloning Ykt6 mutants from eGFP-Ykt6 plasmids using KpnI and MfeI into the mCherry-C1 (a kind gift of Dr. Gregory Smith, Northwestern University). Split Venus constructs (a kind gift from Dr. Michael Schwake, Northwestern University). To generate the Longin domain fraction, the following primers were used: Forward 5'-AACCGGTATGAAGCTGTACAGCCTCAGC-3' and Reverse 5'-TTTTCTCGAGTCAAGCTTCTCGTGGGTTCTG-3'. The PCR product for the Longing domain was gel purified, cut with AgeI and XhoI restriction enzymes, and ligated into the N-terminal Venus construct cut with the same

restriction enzymes. To generate the SNARE domain constructs, human Ykt6 and its mutants described above were used as templates. The following primers sequences were used: Forward 5'-AACCGGTGATCCCATGACTAAAGTGCAG-3' and Reverse primers 5'-TTTTCTCGAGTCATGAGTTTTGTTTCCGGGC-3'. The SNARE domain PCR products were cut with AgeI and XhoI restriction enzymes and were ligated into the C-terminal Venus construct cut with the same restriction enzymes. eGFP-Ykt6 viruses were made by subcloning eGFP-tagged human Ykt6 and its mutants using NheI and NotI and ligated into the lentiviral vector pER4. The following primer sequences were used for PCR amplification of Ykt6/restriction sites: Forward 5'- AAAGCTAGCATGGTGAGCAAGGGCGA-3' and Reverse primer 5'- TGCGGCCGCTCACATGATGGCACAGCAT-3'.

Antibodies

For immunofluorescence the following primary antibodies were used: GFP (Santa Cruz, sc-9996) and TGN46 (Thermo, pa 1-1069). Secondary antibodies used are the following: Alexa 488 (Invitrogen, a21202 and a21206), Alexa 594 (Invitrogen, a21442) and Alexa 647 (Invitrogen, a281883). For Western Blot the following primary antibodies were used: GFP (Santa Cruz, sc-9996), actin (Abcam, ab6276), Ykt6 (SantaCruz, sc-365732), LC3B (Cell Signaling, #2775), p62 (Sigma, p0067), STX17 (Sigma, hpa001204), SNAP29 (Abcam, ab138500) Na+/K+ ATPase (Sigma, 06-172-I), alpha/beta-Tubulin (Cell Signaling, 2148S), and STX7 (Bethyl, A304-512A). Secondary antibodies used are the following: IRDye680 (Fisher, 925-68070) and IRDye800 (Fisher, 925-32211). Secondary antibodies used are the following: IRDye680 (Fisher, 925-68070) and IRDye800 (Fisher, 925-32211).

SDS-PAGE/Western blotting and Immunoprecipitations

Transiently transfected HEK293T cells (18-24 hours post transfection) were briefly washed with ice-cold 1X PBS and lysed using a radioimmunoprecipitation (RIPA) assay buffer (50 mM Tris/HCl pH 7.6; 150 mM NaCl; 20 mM KCl; 1.5 mM MgCl2; 1% NP40; 0.1% SDS) for western blot analysis or 1% Triton X-100 lysis buffer (1% Triton X-100, 10% glycerol, 10 mM Tris/Cl pH 7.5; 150 mM NaCl; 0.5 mM EDTA) for immunoprecipitation experiments. In all experiments, lysis buffer was supplemented with the Halt protease and phosphatase inhibiter cocktail

(Thermofisher; 78441). Samples were incubated on ice for 30 minutes and pushed through a 27G needle (10 times) to ensure full lysis. Samples were then centrifuged at max RPM (~20,000 x g) for 20 minutes. Protein concentration was analyzed with the Pierce BCA Protein Assay kit (Thermofisher). For western blot analysis, after the addition of the appropriate amount of the 6X Laemmli Sample Buffer (Bioland scientific LLC, sab03-02) with 5% ß-mercaptoethanol (Sigma) protein samples were boiled and separated on precast 4-20% Criterion TGX Stain-free gels (Bio-Rad) and transferred to a nitrocellulose membrane (Amersham Protran 0.2 µm NC, #10600001). Membranes were blocked with 5% non-fat milk in 1X Tris-buffered saline (TBS) (50mM Tris/Cl pH 7.4, 150mM NaCl) for 1 hour at room temperature. Membranes were subsequently immunoblotted overnight in primary antibody at 4°C, shaking. The following day, membranes were washed three times with 1X TBST (TBS with 0.1% Tween) for 5 minutes and incubated in secondary IRDye antibody for 1 hour shaking at room temperature. Membranes were washed three times with 1X TBST for before imaging using Li-Cor Odyssey® CLx Imaging System. Images were processed using Image Studio Software (LI-COR Biosciences) and densitometries were quantified using Fiji (51). Before final presentation, resulting densitometry values were normalized to the “control”. Autophagic flux is defined as LC3-II (Baf-A1) minus LC3-II (without Baf-A1). GFP-tagged proteins were immunoprecipitated using the GFP-Trap Agarose® beads (Chromotek; gta-20) according to the manufacture’s protocol and eluted with 2x Laemmli Sample Buffer (BioRad; 1610737) with 5% ß-mercaptoethanol for the subsequent western blot analysis. For the MS analyses, immunoprecipitated proteins were eluted first with elution buffer I (50 mM Tris/Cl pH 7.5, 2 M Urea, 1 mM DTT) by incubating samples in a thermomixer at 30°C for 30 min and shaking at 400 rpm. Supernatant was collected by spinning at 2500 x g at 4°C for 2 min. Second elution was done with elution buffer II (50 mM Tris/Cl pH 7.5, 2 M Urea, 5 mM iodoacetamide) and repeated once. All collected supernatants were combined and incubated in the thermomixer at 32°C at 400rpm overnight, protected from light. Next day, all samples were submitted for MS on the following day.

Partial Proteolysis

HEK293T cells were immunoprecipitated using GFP-Trap beads according to the manufacture's protocol. After the final spin, the supernatant was discarded and a trypsin (Promega; v5111) solution (25mM Tris pH 8; 100mM NaCl; 10% Glycerol; 5mM MgCl2; Sequence Grade Modified Trypsin) at indicated trypsin concentrations was added to bound-beads and incubated at 25°C for 1 hour. Proteolysis was stopped with a stop solution (500nM EDTA; 150mM PMSF; 6X Laemmli Sample Buffer to 400μL). Samples were boiled and half of the samples was run on the precaste4-20% Criterion TGX Stain-free gels (Bio-Rad) and stain with a Pierce Silver Stain kit (Thermo, 24612) and the second half was analyzed by western blot as described above. Silver stained gels were images using Canon CanoScan 9000F scanner.

Mass Spectrometry

Relative phosphopeptide quantification by label-free shotgun proteomics was performed in collaboration with Paola Picotti at ETH Zurich as previously described [5]. Briefly, differentially phosphorylated peptides, we compared control and α-syn-expressing yeast cells. We focused on those peptides showing at least a two-fold (log2) change in abundance with a false discovery rate-q value <0.05. Since calcineurin is a phosphatase highly activated in α-syn-expressing yeast cells, we concentrated our analysis on the two-fold hypo-phosphorylated subset (527 of 5,250) to define phosphosites that may be dephosphorylated by calcineurin/FKBP12. Human wild-type (WT) GFP-Ykt6 phospho-peptides were analyzed by isobaric tag for relative and absolute quantification (iTRAQ) MS was performed by the MIT Koch core facility. Human Ykt6 WT and phosphomutant interactors were analyzed by the tandem mass spectrometry in Jeffrey Savas' laboratory at Northwestern University as previously described [6]. Hits were scored as positive based on the following criteria: 1) present in all the 3 MS analyses, 2) spectral counts had to be >5 for either Ykt6 WT and/or phosphomutants if the GFP control pulldown contained zero spectral counts, and/or 3) spectral counts had to be >10-fold higher for Ykt6 WT and/or phosphomutants if the GFP pulldown control contained greater than zero spectral counts. Sequence alignment was done using Clone Manager software (v 9, Sci-ED software) and sequences were obtained using Uniprot (https://www.uniprot.org/).

Immunofluorescence and live-cell imaging

HeLa cells were seeded on glass coverslips and fixed with 3% formaldehyde (Fisher 04018-1) 20-24 hours post-transfection for 20 minutes at room temperature. Cells were then permeabilized using permeabilization buffer (10% FBS in 1X PBS + 0.1% fresh saponin) for 1 hour at room temperature. Samples were then incubated in primary antibody diluted in permeabilization buffer overnight at 4°C. Coverslips were then washed three times with 1X PBS for 5 minutes each wash. Secondary antibodies corresponding to the primary antibodies were diluted in permeabilization buffer and added to the samples for 1 hour at room temperature. Samples were washed three times with 1X PBS for 5 minutes each wash and then mounted using a DAPI-staining mounting medium (Vector Laboratories; H-1200-10). Cells were imaged using Leica DMI4000B microscope fitted with Leica TCS SPE laser-scanning confocal system with Solid State lasers (405/488/561/635) and the 40X/1.15 OIL CS ACS APO objective. XY and Z images (10 step, 0.67 micron step) were captured using Leica Application Suite X (LAS X) software. For live-cell imaging, Leica DMI3000B microscope fitted with an QImaging QIClick CCD Camera and Leica HCX PL FLUOTAR L 40X/0.60 CORR PH2 objective was used. Transiently transfected HeLa cells were imaged in VALAB (Vaseline, lanolin, and beeswax) sealed chambers as described previously [7]. Image acquisition was done using Q-capture pro7 software and manual tracking of the equal exposure and digital gain setting between images. All image processing and analysis was done using Fiji [8]. All experiments were done three independent times and analyzed in a randomized and blinded fashion. GFP-LC3-II and split Venus integrated fluorescent intensity was analyzed using CTCF (Corrected Total Cell Fluorescence) = Integrated Density - (Area of selected cell X Mean fluorescence of background readings) formula and normalized to the CTCF value in control cells (fold over control). For the colocalization analysis, Mender's coefficient was calculated using coloc2 plugin in Fiji. Autophagic flux is defined as LC3-II (Baf-A1) minus LC3-II (without Baf-A1). All values were normalized to the control condition and presented as fold change.

Structural Analysis

The phosphorylated versions were obtained using Pymol plugin Pytms 52 by modification of the structure of the closed conformation of rat Ykt6 (PDB code 3KYQ) resolved by X-ray resolved diffraction. Model quality was

assessed with Molprobity [9]. According to the evaluation, the generated models have been placed in 100th percentile, arguing that the geometry and contacts of amino acids within the models are comparable to those of the finest current structures in Protein Data Bank. Kinimages visualizing the detailed Molprobity report were generated with KiNG (v2.21). The open conformational state was created by adjusting torsion angles of the backbone in the region 162-164 amino acids using Chimera v1.13 54. Electrostatic potential was computed with APBS plugin of Chimera package. Sequence conservation was analyzed using 196 fungal and 140 animal sequences of Ykt6 proteins retrieved from Tracey database [10]. Sequence logos were created using WebLogo generator [11].

Cell fractionation

HEK293T cells (DMEM, 10 % FBS, 1X P/S) were seeded to ~80 % confluency a day prior to the experiment in a 10 cm dish. Cells were transfected with empty vector (pEGFP-C1), GFP-tagged- human YKT6 wild type, -S174A and -S174D variants using JetPrime exactly as per manufacturer's recommendation. Media was replaced after 6 hours and cells were allowed to express the proteins for 24 hours. Subcellular fractionation of transfected cells was carried out exactly as described[12]. Fifteen micrograms of the total cell, membrane and cytoplasmic fractions were separated on SDS-PAGE and probed with mouse anti-GFP antibody (Santacruz, 1:2000). Blots were also probed with rabbit anti-Na+/K+ ATPase α-3 antibody (Millipore Sigma, 1:1000) and rabbit anti-tubulin (Cell signaling, 1:10,000) to assess the purity of the membrane and cytoplasmic fractions, respectively. IRDye 800CW goat anti-rabbit and IRDye 680 goat anti-mouse were used as secondary antibodies (Li-COR, 1:10,000). Blots were developed using Odyssey CLx imaging system (LI-COR Biosciences).

Protein expression and purification

Construction of 6XHis-tagged YKT6 variants. The genes encoding the wild type-, S174A- and S174D-YKT6 were PCR amplified using human eGFP-WT/S174A-YKT6/S174D-YKT6 constructs as the templates, respectively and the primers (Forward: 5′- TACTTCCAATCCAATATGAAGCTGTACAGCCTCAGCG-3′; Reverse: 5′-TTATCCACTTCCAATTGAGTTTTGTTTCCGGGC-3′). The PCR products were cloned by ligation independent

cloning in the pMCSG53 vector (1) (which encodes N-terminal 6xHis-tag with a tobacco etch virus (TEV) protease cleavage site) using CloneAmp HIFi PCR Premix (InFusion HD plus systems) according to the manufacturer's instructions. We amplified only 1-579 bp of the gene and excluded the 15 bp from the C-terminal end encoding five amino acids that are involved in the post-translational modification (palmitoylation and farnesylation) of this protein in the mammalian cells. Putative recombinants were screened using restriction enzyme digestion using NdeI/BamHI. The integrity of the YKT6 gene in the constructs was confirmed by DNA sequencing.

The plasmids (WT-YKT6ΔCCAIM, S174A-YKT6ΔCCAIM and S174D-YKT6ΔCCAIM) were transformed into kanamycin resistant competent cells *E. coli* BL21(DE3)(pMagic) (2) for the expression analysis. The expression of the recombinant protein was analyzed in the minimal M9 medium supplemented with the labelled isotopes-^{15}N-ammonium sulfate and ^{13}C-U6-D-glucose (Cambridge Isotope Laboratories, Inc). The protein expression was induced by addition of 1 mM Isopropyl β-d-1-thiogalactopyranoside (IPTG) (Sigma) at OD_{600} of 0.8-1.0 for ~18 hours at 25 °C with 220 rpm agitation. The cells expressing the recombinant proteins were re-suspended in the lysis buffer (50 mM Tris HCl, pH 7.5, 500 mM NaCl, 0.1 % IGEPAL CA-630 [13], 20 % glycerol, 1 X protease inhibitor cocktail-EDTA free {Thermofisher}). The pellets were stored at −80°C until purification. The bacterial suspension was thawed and subjected to sonication using a pulse of 5 sec on 10 sec off during 20 min. The lysate was spun at 14000 rpm for 45 min at 4 °C, supernatant was collected and used for protein purification. Briefly, the clarified lysate was loaded onto a His-Trap FF (Ni2+NTA) column using a GE Healthcare AKTA Purifier system in loading buffer (10 mM Tris-HCl pH 7.5, 500 mM NaCl, 20 % glycerol, 1 mM Tris(2-carboxyethyl)phosphine (TCEP) (Sigma). The column was washed with high salt buffer (10 mM Tris-HCl pH 7.5, 1 M NaCl, 20 % glycerol, 1 mM TCEP, 25 mM imidazole) and the protein was eluted with high imidazole buffer (10 mM Tris-HCl pH 7.5, 500 mM NaCl, 20 % glycerol, 1 mM TCEP, 1 M imidazole). The eluent was loaded into a Superdex 200 26/600 column equilibrated with loading buffer. The protein fractions were pooled and the 6x-His-tag was removed using TEV protease. The protease and the recombinant protein were mixed in 1:20 ratio (1 mg TEV/20 mg protein) and incubated at room temperature for overnight (~18 hrs). The untagged protein was separated from the tagged protein and the TEV protease by loading the sample on the Ni2+NTA column. Most

of the cleaved protein was recovered from the column by adding 200 mM imidazole. The purified protein fractions were pooled and dialyzed in the NMR buffer (10 mM Tris-HCl, pH 6.8, 100 mM NaCl). The protein was concentrated using a 10 kDa MWCO Vivaspin 20 (GE healthcare) to desired concentration (0.1 mM for 2D-NMR and 0.5 mM for 3D-NMR) The protein concentration was estimated on a nanodrop using molar extinction coefficient of YKT6 to be 18910 M-1cm-1. Protein aliquots were snap frozen in liquid nitrogen until further use. Samples were submitted to the University of Wisconsin, National Magnetic Resonance Facility at Madison (NMRFAM).

NMR

a. Preparation of NMR samples

For chemical shift assignments, freshly prepared samples 13C/15N -labeled Ytk6 were exchanged into a solution containing 10 mM Tris buffer, pH 6.8, 100 mM sodium chloride, 0.05% sodium azide, 1X protein inhibitor cocktail (Sigma–Aldrich) and 10% D2O (Sigma–Aldrich); the final concentration of each protein was 0.640 mM. Samples used for NMR spectroscopy (300 µL) were enclosed in 5-mm susceptibility-matched Shigemi NMR tubes (Shigemi, Allison Park, PA). For S174A and S174D mutants of Ytk6, 15N-labeled samples were exchanged into the same buffer as for the wild-type. The final concentration of the proteins was 0.128 mM and 0.270 mM for the S174A and S174D mutants, respectively. Samples of 120 µL of volume were used with 3-mm NMR tubes to record spectra for these mutant proteins.

b. NMR data collection.

Titration of Ytk6 with DPC. Ytk6 bound to dodecylphosphocholine (DPC) was used to record spectra for backbone assignments. DPC was titrated into a solution of 13C/15N -labeled Ytk6. The binding of DPC was monitored by inspecting chemical shift changes of the Ytk6 peaks in 2D ^{1}H,^{15}N-HSQC spectra recorded after each addition of DPC. The titration was stopped when the position of the peaks stopped

changing, indicating that the protein has been saturated with DPC. The ratio of DPC to Ytk6 at the end of the titration was approximately 2:1. The 2D $^{1}H,^{15}N$-HSQC spectra used to follow the titration were recorded on a Bruker Avance III spectrometer operating at 600 MHz (1H) equipped with a cryogenic triple-resonance probe. Sample temperature was regulated at 298°K.

c. Backbone assignments of Ytk6

All NMR spectra on the $^{13}C/^{15}N$ -labeled Ytk6 sample bound to DPC were acquired on Bruker Avance III or Varian VNMRS spectrometers operating at 900 and 600 MHz (1H); each was equipped with a cryogenic triple-resonance probe. The temperature of the sample was regulated at 308°K for all experiments.

For backbone assignments 2D $^{1}H,^{15}N$-HSQC and 3D HNCACB, 3D CBCA(CO)NH, and 3D HNCA spectra were collected using the acquisition parameters reported in Table 1.

All 3D spectra were recorded using non-uniform sampling (NUS) with a sampling rate of ~35%. In addition, the 3D HNCA spectrum was recorded using band-selective excitation short-transient sequences (BEST-type experiments) with a recovery delay of 0.4 s between transients [14]. All spectra were processed using NMRPipe [15], and the 3D spectra recorded using NUS were reconstructed and processed using the SMILE package [15], included with NMRPipe. The spectra were analyzed using the NMRFAM-SPARKY software package [16]. The peaks were initially picked using the APES automation plugin [17] and verified manually. The peak lists were then submitted to I-PINE [18] for automated assignments of the backbone resonances. The assignments by I-PINE were then verified by hand in NMRFAM-SPARKY.

d. Ytk6 mutants

DPC was added to the samples of ^{15}N-labeled S174A and S174D Ytk6 to achieve a final DPC to protein ratio of 2 to1. 2D ^{1}H,^{15}N-HSQC spectra on the Ytk6 mutants bound to DPC were acquired on an Avance III Bruker spectrometer operating at 600 MHz (1H) and equipped with a cryogenic triple-resonance probe. The temperature of the samples was regulated at 308°K. All spectra were processed using NMRPipe and inspected with NMRFAM-SPARKY. The backbone ^{1}H,^{15}N assignments were transferred from the spectrum of wild-type Ytk6 and the position of the peak markers was adjusted by hand to match the peaks in the spectra of the mutants. The chemical shift perturbation (CSP) between mutants and wild-type Ytk6 was calculated using the “NMR perturbation plot” extension (np) in NMRFAM-SPARKY by applying the formula: CSP=√(2&[〖ΔH〗^2+(〖ΔN〗^2/B)]/2) where ΔH and ΔN are the change in 1H and 15N chemical shift, respectively, between corresponding mutant and wild-type Ytk6 peaks, and B=5 is the scaling factor used for the ^{15}N chemical shits.

Statistical Analysis

GraphPad Prism 7 Software (http://www.graphpad.com) was used to graph, organize, and perform all statistical analyses. Values are expressed as an average + the standard error mean (SEM). Statistical analysis was determined using the following methods: One-way analysis of variance (ANOVA) with either Tukey’s test or Fisher’s uncorrected LSD test. Two-way ANOVA with Fisher’s uncorrected LSD test. P-values <0.05 were considered significant. A minimum of three biological replicates were used for each experiment. The specific number of biological replicates for each experiment is listed in the Figure Legends.

C. elegans methods

C. *elegans* Strain Construction

Nematodes were grown and maintained on OP50-1 E. coli bacteria at 20°C under standard laboratory conditions (Brenner, 1974). Plasmids were created as follows: C. elegans *ykt-6* cDNA (accession# NM_066204.5) was

obtained from GenScript (Piscataway, NJ). Site-directed mutation in *ykt-6* (S177A and S177D) constructs were generated using Q5 mutagenesis kit (Bioengland labs) to mutate serine 177 of ykt-6 in pcDNA3.1+C-(K)-DYK vector (Genscript). Using primers containing attB sites, WT and mutated *ykt-6* was amplified from pcDNA3.1+C-(K)-DYK vector. Amplified DNA was cloned into pDONR221 via BP reaction to generate entry clones of WT and mutated *ykt-6*. Using LR reaction of entry clones and pDEST-DAT1 vector (Cao et al., 2005), expression clones of WT and mutated *ykt-6* were generated and confirmed by sequencing. Gateway entry vectors were verified by DNA sequencing to validate the constructs.

<u>Primers used to mutagenize Ykt6 phosphosite (S177) on pcDNA3.1+C-(K)-DYK-ykt6 (5'-3')</u>

Forward primer for change to alanine: AGTGAAAAAGGCTGAGAACCTGTCGG

Forward primer for change to aspartate: AGTGAAAAAGGATGAGAACCTGTCGG

Common reverse primer: AAATCATCCAGCTTCTCAC

<u>Primers used to amplify *ykt-6* from pcDNA3.1+C-(K)-DYK-ykt6 with attB arms for BP reaction with pDONR221 (5'-3')</u>

Forward primer:

GGGGACAAGTTTGTACAAAAAAGCAGGCTCCATGAAATTATACTCAATTCTTGTATTTCACAAAAACGTCG

Reverse primer:

GGGGACCACTTTGTACAAGAAAGCTGGGTCTTAGACGTAATTGCAGCATTTGTTCATCTTTCGAGC

<u>Primers used to sequence verify expression clones (5'-3')</u>

Forward primer: CCCGCTACGGTTTACTCG

Reverse primer: TTGGACTTAGAAGTCAGAGG

Q5 mutagenesis kit was used to mutate serine 177 of *ykt-6* in pcDNA3.1+C-(K)-DYK vector (Genscript). Using primers containing attB sites, WT and mutated *ykt-6* were amplified from pcDNA3.1+C-(K)-DYK vector. Amplified DNA was cloned into pDONR221 via BP reaction to generate entry clones of WT and mutated *ykt-6*. Using LR reaction of entry clones and pDEST-DAT1 vector, expression clones of WT and mutated *ykt-6* were generated and confirmed by sequencing.

Primers used to mutagenize *ykt-6* phosphosite (S177) on pcDNA3.1+C-(K)-DYK-ykt6 (5'-3')Forward primer for change to alanine: AGTGAAAAAGGCTGAGAACCTGTCGG

Forward primer for change to aspartate: AGTGAAAAAGGATGAGAACCTGTCGG

Common reverse primer: AAATCATCCAGCTTCTCAC

Primers used to amplify *ykt-6* from pcDNA3.1+C-(K)-DYK-ykt6 with attB arms for BP reaction with pDONR221 (5'-3')

Forward primer:

GGGGACAAGTTTGTACAAAAAAGCAGGCTCCATGAAATTATACTCAATTCTTGTATTTCACAAAAACGTCG

Reverse primer:

GGGGACCACTTTGTACAAGAAAGCTGGGTCTTAGACGTAATTGCAGCATTTGTTCATCTTTCGAGC

Primers used to sequence verify expression clones (5'-3')

Forward primer: CCCGCTACGGTTTACTCG

Reverse primer: TTGGACTTAGAAGTCAGAGG

All transgenic strains were created by injecting expression plasmids into N2 Bristol strain using previously described methods (Berkowitz et al., 2008). The co-injection marker for all transgenic strains was P_{unc-54}::tdTomato. Both the expression clones and the co-injection marker were injected together at a concentration of 50ng/µl each for the creation of the transgenic strains. As an initial control, the empty P_{dat-1} destination vector was injected into *C. elegans* to determine if extra copies of P_{dat-1} promoter/ *unc-54* UTR had an effect by itself. The transgenic lines in the N2 background (3 independent stable lines of each) were then crossed with both the UA44 (baIn11[P_{dat-1}::α-syn, P_{dat-1}::GFP]) and BY250 (vtIs7[P_{dat-1}::GFP]) (gift from Dr. Randy Blakely) strains. The resulting strains created for use in this study were: UA395 (vtIs7[P_{dat-1}::GFP]; baEx219[P_{dat-1} DEST, P_{unc54}::tdTomato]), UA396 (vtIs7[P_{dat-1}::GFP]; baEx220[P_{dat-1}::*ykt-6* WT, P_{unc54}::tdTomato]), UA397 (vtIs7[P_{dat-1}::GFP]; baEx221[P_{dat-1}::*ykt-6* S177D, P_{unc54}::tdTomato]), UA398 (vtIs7[P_{dat-1}::GFP]; baEx222[P_{dat-1}::*ykt-6* S177A, P_{unc54}::tdTomato]), UA399 (baIn11[P_{dat-1}::α-syn, P_{dat-1}::GFP]; baEx219[P_{dat-1} DEST, P_{unc54}::tdTomato]), UA400 (baIn11[P_{dat-1}::α-syn, P_{dat-1}::GFP]; baEx220[P_{dat-1}::*ykt-6* WT, P_{unc54}::tdTomato]), UA401 (baIn11[P_{dat-1}::α-syn, P_{dat-}

$_{1}$::GFP]; baEx221[P$_{dat\text{-}1}$::*ykt-6* S177D, P$_{unc54}$::tdTomato]), UA402 (baIn11[P$_{dat\text{-}1}$::α-syn, P$_{dat\text{-}1}$::GFP]; baEx222[P$_{dat\text{-}1}$::*ykt-6* S177A, P$_{unc54}$::tdTomato]).

Dopaminergic Neurodegeneration Analysis

Worms were synchronized to be as close to the same age as possible by performing 3-hour egg lays for all experiments. All worms were kept at 20°C for the entire duration of each experiment. Great care was used to ensure that only transgenic worms were used for these analyses, which were selected for via tdTomato expression. The extent of neurodegeneration for all experiments was determined on Days 4 and 6 post egg lay. The following was performed for each replicate: worms were placed in a 6 µL drop of 10mM levamisole (dissolved in 0.5X S basal buffer) on a glass coverslip. This drop of levamisole on the coverslip was inverted and placed on the surface of a 2% agarose pad, made on a microscope slide. The 6 dopaminergic neurons in the head region of worms were then observed and scored for the extent of neurodegeneration via fluorescence microscopy. Each worm was categorized as either normal or degenerative. Worms were categorized as degenerative if at least 1 out of the 6 dopaminergic neurons in the head region exhibited a break in the dendritic processes and/or an absence or abnormality of a cell body. Worms were only categorized as normal if both cell bodies and dendritic processes were completely intact. All experiments consisted of 3 separate biological replicates (of 3 separate stable lines). Each biological replicate consisted of 1 stable line of each strain, each consisting of 3 technical replicates. Each technical replicate consisted of 30 worms (30 worms x 3 technical replicates = 90 worms per biological replicate). One-Way ANOVA using a Tukey's post hoc test was performed using GraphPad Prism Software for determining statistical significance among groups.

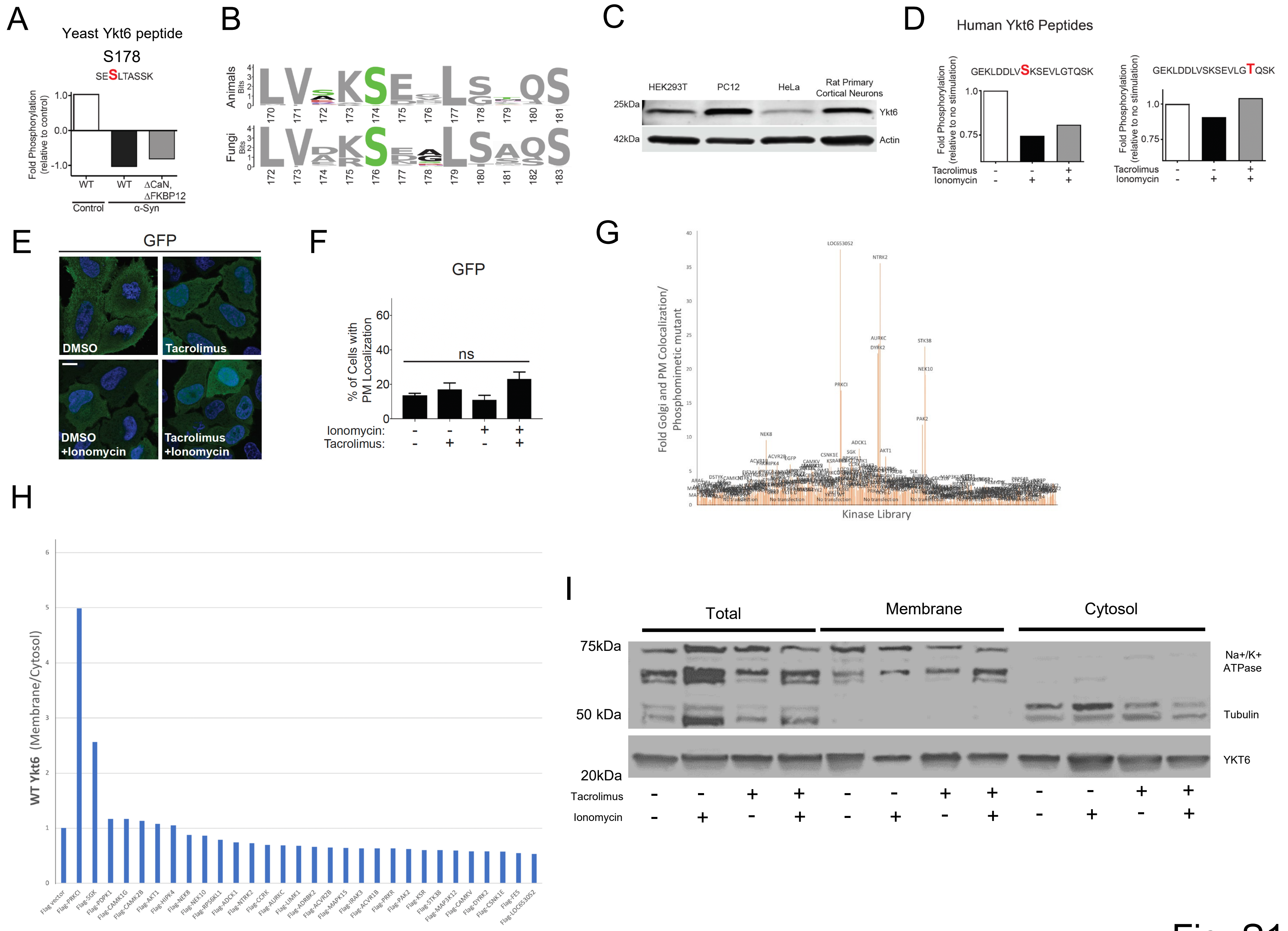
A
Yeast Ykt6 peptide
S178
SESLTASSK
Fold Phosphorylation (relative to control)
1.0
0.0
-1.0
WT
WT
ΔCaN, ΔFKBP12
Control
α-Syn
B
Animals
Fungi
Bits
C
HEK293T
PC12
HeLa
Rat Primary Cortical Neurons
25kDa
42kDa
Ykt6
Actin
D
Human Ykt6 Peptides
GEKLDDLVSKSEVLGTQSK
GEKLDDLVSKSEVLGTQSK
Fold Phosphorylation (relative to no stimulation)
Tacrolimus
Ionomycin
E
GFP
DMSO
Tacrolimus
DMSO +Ionomycin
Tacrolimus +Ionomycin
F
GFP
% of Cells with PM Localization
ns
Ionomycin:
Tacrolimus:
G
Fold Golgi and PM Colocalization/ Phosphomimetic mutant
Kinase Library
LOC653052
NTRK2
AURKC
DYRK2
STK38
NEK10
PRKCI
PAK2
NEK8
ADCK1
AKT1
SGK
H
WT Ykt6 (Membrane/Cytosol)
Flag-vector
Flag-PRKCI
Flag-SGK
Flag-PDPK1
Flag-CAMK1G
Flag-CAMK2B
Flag-AKT1
Flag-HIPK4
Flag-NEK8
Flag-NEK10
Flag-RPS6KL1
Flag-ADCK1
Flag-NTRK2
Flag-CCRK
Flag-AURKC
Flag-LIMK1
Flag-ADRBK2
Flag-ACVR2B
Flag-MAPK15
Flag-IRAK3
Flag-ACVR1B
Flag-PRKR
Flag-PAK2
Flag-KSR
Flag-STK38
Flag-MAP3K12
Flag-CAMKV
Flag-DYRK2
Flag-CSNK1E
Flag-FES
Flag-LOC653052
I
Total
Membrane
Cytosol
75kDa
50 kDa
20kDa
Na+/K+ ATPase
Tubulin
YKT6
Tacrolimus
Ionomycin

Fig. S1

Fig.S1. (A) Fold phosphorylation of the indicated peptide from endogenous yeast Ykt6 detected by shotgun phosphoproteomics after correction for protein abundance from control yeast cells and yeast cells with high levels of Ca^{2+} (driven by overexpression of α-syn) with either WT or knockout for calcineurin (ΔCaN) and knockout for the modulator of calcineurin (ΔFKBP12). The identified phosphorylation sites are highlighted in red. Data from triplicate samples was pulled together for illustrated analysis[28]. **(B)** Animal and fungal Ykt6 protein sequences by sequence logos obtained from Tracey database and aligned. The residues associated with evolutionary conserved positions are shown. The CaN-dependent phosphorylation sites retrieved from the mass spectrometry screens are shown in green. **(C)** Representative western blot for Ykt6 expression in different cell types: HEK293T, PC12, HeLa and rat primary cortical neurons. Actin serves as a loading control. **(D)** Fold phosphorylation of the indicated human Ykt6 peptides from HEK293T cells as detected by iTRAQ mass spectrometry. Prior to GFP-Ykt6 immunoprecipitation, cells were treated for 30 minutes with the Ca^{2+} ionophore ionomycin (1μM) and co-treated with calcineurin-specific inhibitor Tacrolimus (1μM). **(E)** Representative immunofluorescence images of transiently transfected HeLa cells with GFP. Cells were treated with ionomycin (1μM) and/or Tacrolimus (1μM) for 30 minutes. Nuclei (blue) are stained with DAPI. Scale bar is 10μm. **(F)** Quantification of cells with GFP plasma membrane localization as shown in (A). N=3 *$p<0.05$ ***$p<0.001$ ****$p<0.0001$ One-way ANOVA, uncorrected Fisher's LSD test**. (G)** Fold Golgi and plasma membrane (PM) colocalization of the 300 clones tested from the human kinome Flag tagged Gateway in the presence of GFP WT Ykt6. Kinase hits were considered positive as those 5 fold above the colocalization signal from the phosphomimetic mutant (S174D). **(H)** The 30 kinase hits passing the criteria described in (G) were selected and individually tested. A line-scan fluorescence correlation was used to further score the hits. Positive hits were considered above the line-scan correlation from GFP- WT Ykt6 with Flag empty vector. **(I)** Representative western blot of 3 after cell fractionation for endogenous Ykt6 in HEK293T cells after the indicated pharmacological treatment as in (D). Membrane fraction is defined by Na+K+ and cytosolic fraction is defined by Tubulin.

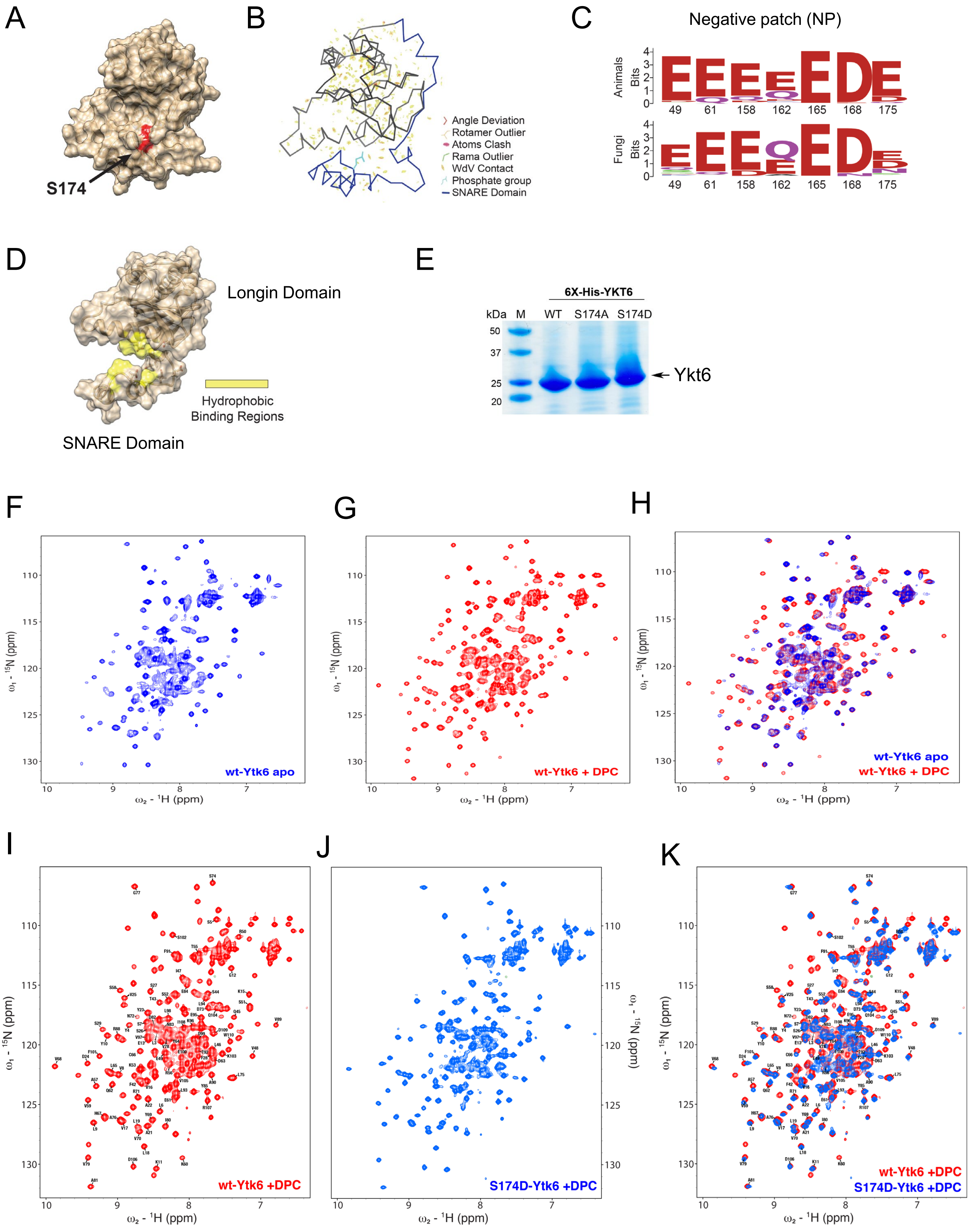
A
S174
B
Angle Deviation
Rotamer Outlier
Atoms Clash
Rama Outlier
WdV Contact
Phosphate group
SNARE Domain
C
Negative patch (NP)
Animals
Fungi
Bits
D
Longin Domain
Hydrophobic Binding Regions
SNARE Domain
E
6X-His-YKT6
kDa M WT S174A S174D
Ykt6
F
wt-Ytk6 apo
G
wt-Ytk6 + DPC
H
wt-Ytk6 apo
wt-Ytk6 + DPC
I
wt-Ytk6 +DPC
J
S174D-Ytk6 +DPC
K
wt-Ytk6 +DPC
S174D-Ytk6 +DPC

Fig. S2

Fig.S2. (A) Surface model of Ykt6 closed conformation with the human evolutionary-conserved CaN-sensitive phosphorylation site highlighted in red. **(B)** Visualization of atom clashes, poor rotamers, torsion angle outliers using Molprobity and KiNG mimicking the phosphorylation of the human evolutionary-conserved CaN-sensitive site in Ykt6 closed conformation with the SNARE domain highlighted in blue. **(C)** Animal and fungal Ykt6 protein sequences were obtained from Tracey database and aligned. The residues corresponding to indicated positions from 3KYQ structure were extracted. Conservation of these residues is shown by sequence logos. **(D)** Surface model of Ykt6 open conformation depicting the residues mediating hydrophobic binding in yellow when in the open conformation. **(E)** Coomasie staining for His tagged purified proteins after Histidine cleavage. **(F)** Two-dimensional $^{1}H,^{15}N$-HSQC spectra recorded on a ^{15}N-labeled sample of Ytk6 alone (apo) and **(G)** after addition of a 2:1 excess of DPC and **(H)** overlaid. **(I)** Two-dimensional $^{1}H,^{15}N$-HSQC spectra recorded on a ^{15}N-labeled sample of Ytk6 with DPC with amino acids mapped. **(J)** Two-dimensional $^{1}H,^{15}N$-HSQC spectra recorded on a ^{15}N-labeled sample of Ytk6 S174D with DPC. **(K)** Overlaid two-dimensional $^{1}H,^{15}N$-HSQC spectra recorded on a ^{15}N-labeled sample of WT and S174D Ytk6 with DPC with amino acids mapped. These spectra were recorded on a Bruker Avance III spectrometer operating at 600 MHz (^{1}H) and equipped with a cryogenic probe. The temperature of the sample was regulated to 25°C.

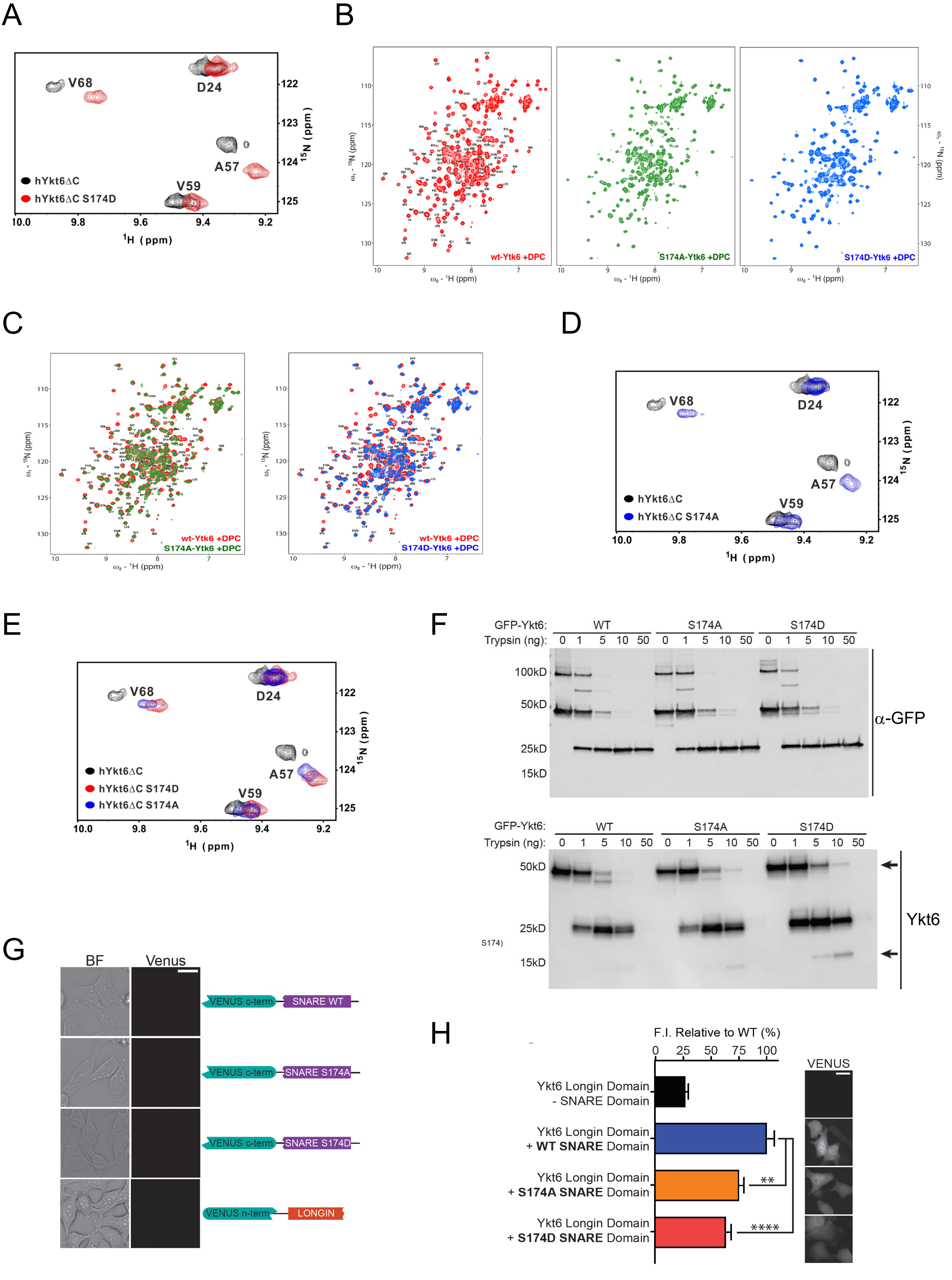
A
V68
D24
A57
V59
hYkt6ΔC
hYkt6ΔC S174D
10.0
9.8
9.6
9.4
9.2
122
123
124
125
1H (ppm)
15N (ppm)
B
wt-Ytk6 +DPC
S174A-Ytk6 +DPC
S174D-Ytk6 +DPC
C
wt-Ytk6 +DPC
S174A-Ytk6 +DPC
wt-Ytk6 +DPC
S174D-Ytk6 +DPC
D
hYkt6ΔC
hYkt6ΔC S174A
E
hYkt6ΔC
hYkt6ΔC S174D
hYkt6ΔC S174A
F
GFP-Ykt6:
WT
S174A
S174D
Trypsin (ng):
0
1
5
10
50
100kD
50kD
25kD
15kD
α-GFP
Ykt6
S174)
G
BF
Venus
VENUS c-term
SNARE WT
SNARE S174A
SNARE S174D
VENUS n-term
LONGIN
H
F.I. Relative to WT (%)
0
25
50
75
100
VENUS
Ykt6 Longin Domain
- SNARE Domain
Ykt6 Longin Domain
+ WT SNARE Domain
Ykt6 Longin Domain
+ S174A SNARE Domain
Ykt6 Longin Domain
+ S174D SNARE Domain
**
****

Fig.S3

Fig.S3. **(A)** ^{15}N-^{1}H TROSY of 0.4 mM 25-kDa Ykt6 at 600 MHz. WT Ykt6 (black) vs S174D (red) in the absence of DPC. **(B-C)** Two-dimensional ^{1}H,^{15}N-HSQC spectra recorded on ^{15}N-labeled samples of WT Ytk6 (red), S174A (green) and S174D (blue). All spectra were recorded in the presence of a 2:1 excess of DPC. The spectra are shown side-by-side (B) and overlaid (C). The peaks for the residues that were able to assign are labeled in the spectrum of WT Ytk6. These spectra were recorded on a Bruker Avance III spectrometer operating at 600 MHz (^{1}H) and equipped with a cryogenic probe. The temperature of the sample was regulated to 35°C. **(D)** ^{15}N-^{1}H TROSY of 0.4 mM 25-kDa Ykt6 at 600 MHz. WT Ykt6 (black) vs S174A (blue) in the absence of DPC. **(E)** same as in (D) including Ykt6 S174D (red). **(F)** Representative western blot immunobloteed for GFP and Ykt6 from GFP-tagged purified WT and phosphomutants of human Ykt6 incubated with the indicated amounts of trypsin for 1 hour at 37°C. Arrows indicate specific cleavage products. **(G)** Representative live-cell phase contrast (BF=Bright Field) and fluorescence images for HeLa cells transiently expressing indicated single split Venus constructs of either Longin domain (LONGIN) alone, and the SNARE domains of either: wild-type (SNARE WT), phosphoablative (SNARE S174A) or phosphomimetic (SNARE S174D) of human Ykt6 alone. Scale bar is 10µm. **(H)** Representative live-cell fluorescence images of HeLa cells transiently expressing with the indicated combinations of split Venus constructs. Quantification of fluorescence intensity of Venus normalized by the area of the cell. Wild-type (WT) Longin domain with the SNARE domains of either: wild-type (SNARE WT), phosphoablative (SNARE S174A) or phosphomimetic (SNARE S174D) of human Ykt6. Scale bar is 10µm. N=4 * $p< 0.05$ One-Way ANOVA, Tukey's test.

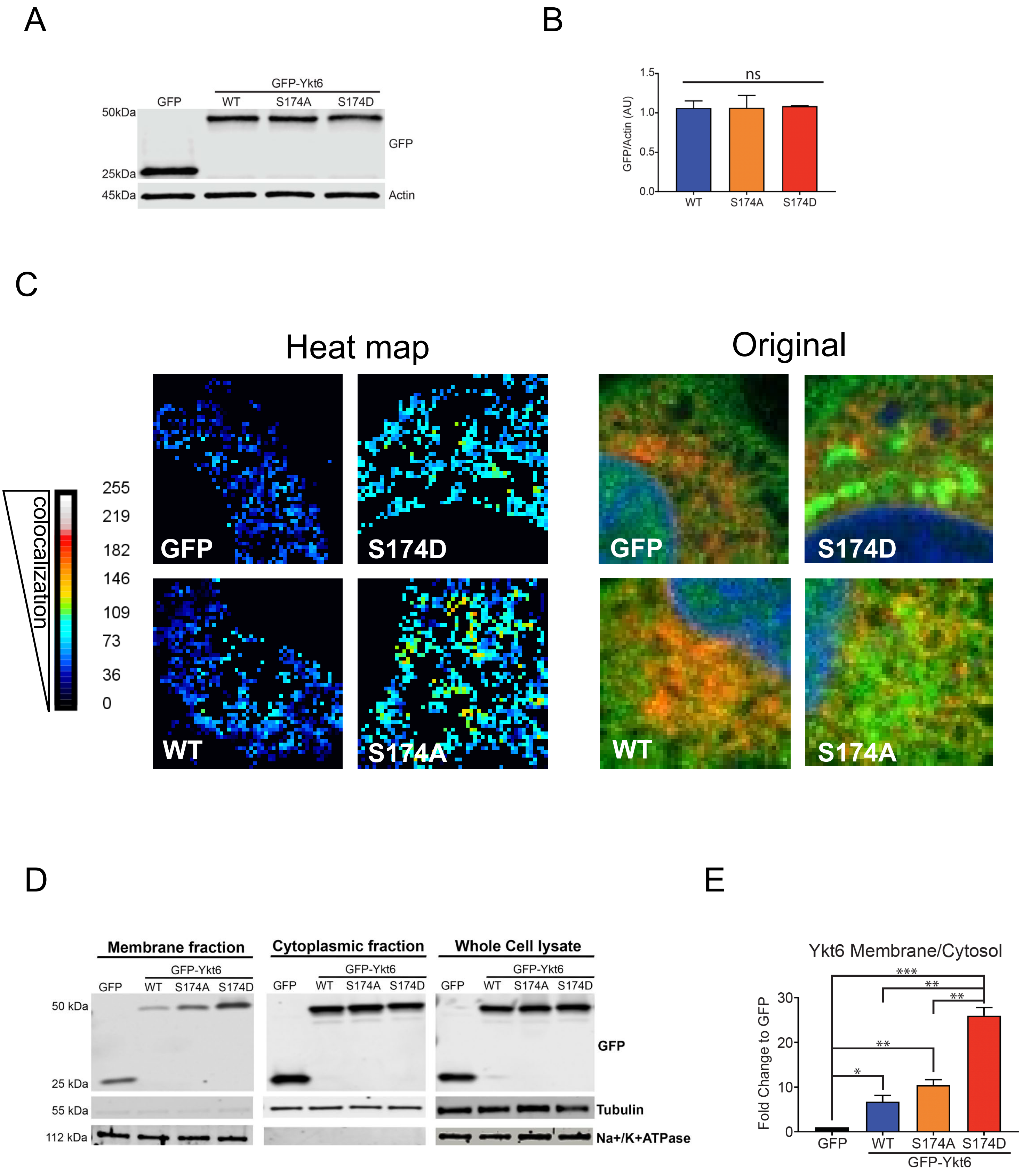
A
GFP-Ykt6
GFP
WT
S174A
S174D
50kDa
25kDa
45kDa
GFP
Actin
B
GFP/Actin (AU)
ns
1.5
1.0
0.5
0.0
WT
S174A
S174D
C
Heat map
Original
colocalization
255
219
182
146
109
73
36
0
GFP
S174D
WT
S174A
GFP
S174D
WT
S174A
D
Membrane fraction
Cytoplasmic fraction
Whole Cell lysate
GFP-Ykt6
GFP
WT
S174A
S174D
50 kDa
25 kDa
55 kDa
112 kDa
GFP
Tubulin
Na+/K+ATPase
E
Ykt6 Membrane/Cytosol
Fold Change to GFP
30
20
10
0
***
**
**
**
*
GFP
WT
S174A
S174D
GFP-Ykt6

Fig.S4

Fig.S4. (A) Representative western blot from transiently transfected HEK293T cells with GFP, GFP-tagged wild-type (WT), phospho-ablative mutant (S174A) and phospho-mimetic mutant (S174D) of Ykt6. **(B)** Densitometry analysis from Western blot from transiently transfected HEK293T cells with GFP, GFP-tagged wild-type (WT), phospho-ablative mutant (S174A) and phospho-mimetic mutant (S174D) of Ykt6 in Figure 2A. Actin serves as loading control. AU=arbitrary units. **(C)** Heat map representation of colocalization analysis of images from fig.3A representing transiently co-transfected HeLa cells with either GFP, GFP-tagged WT or phosphomutants of Ykt6 and mCherry-Sec61 for ER. Nuclei (blue) are stained with DAPI**. (D)** Representative Western Blot for membrane and cytosolic fractions of HEK293T cells transiently transfected as described in (A). Membrane (Na+/K+ ATPase) and cytosolic (tubulin) markers serve as controls for fractionation purity. **(E)** Quantitation of fold-change of membrane/cytosol fraction calculated as: 1) normalizing Ykt6 protein expression (GFP)/actin, 2) obtaining the ratios of GFP-Ykt6 fusion protein to tubulin (for the cytosolic fraction), GFP-Ykt6 fusion protein to Na+/K+ ATPase (for the membrane fraction) and 3) membrane fraction/cytosolic fraction. N=4 *p,0.05, **p<0.01, ***p,0.001 non-parametric *t-test*.

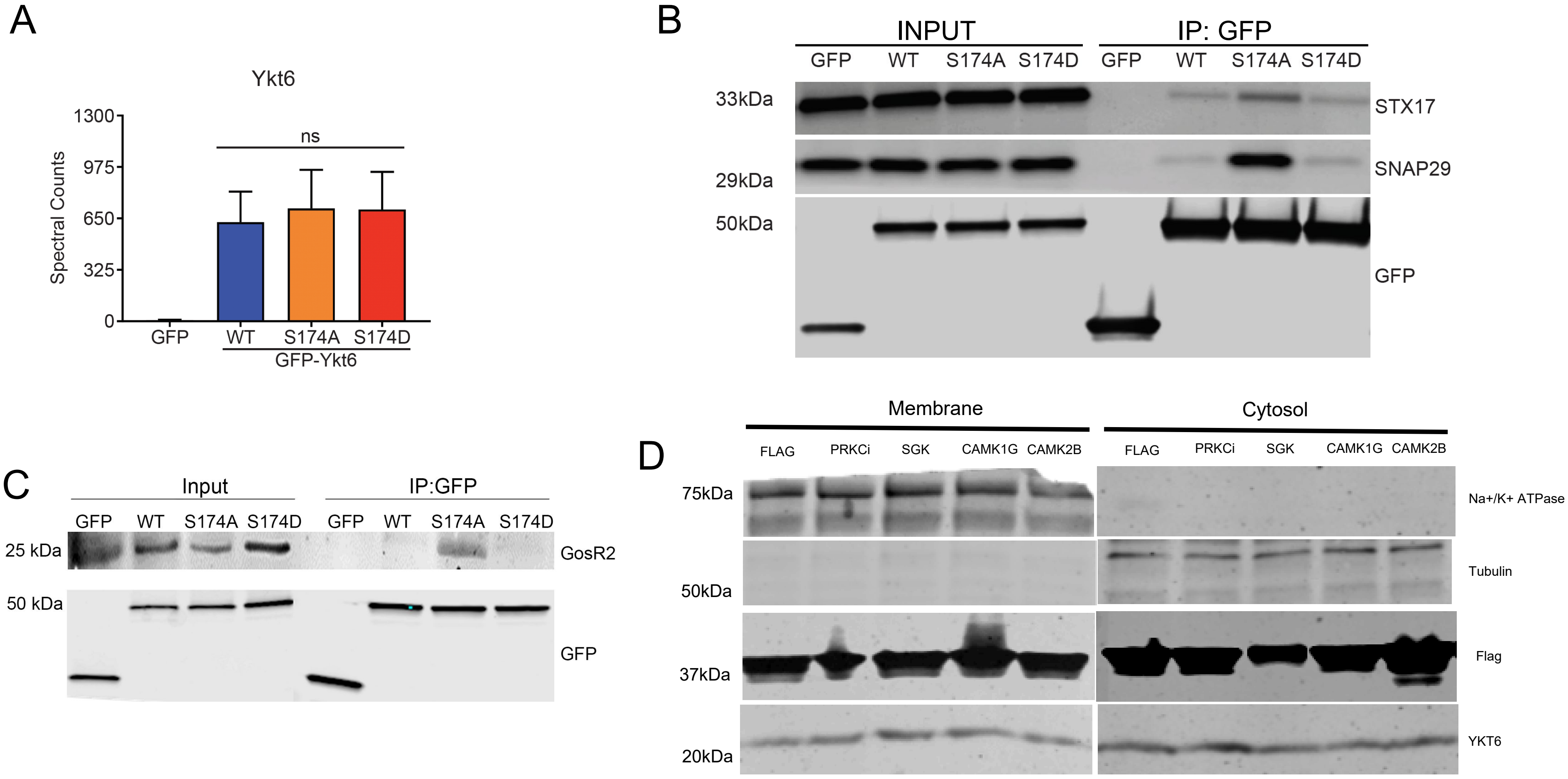
A
Ykt6
Spectral Counts
1300
975
650
325
0
ns
GFP
WT
S174A
S174D
GFP-Ykt6
B
INPUT
IP: GFP
GFP WT S174A S174D GFP WT S174A S174D
33kDa
29kDa
50kDa
STX17
SNAP29
GFP
C
Input
IP:GFP
GFP WT S174A S174D GFP WT S174A S174D
25 kDa
50 kDa
GosR2
GFP
D
Membrane
Cytosol
FLAG PRKCi SGK CAMK1G CAMK2B FLAG PRKCi SGK CAMK1G CAMK2B
75kDa
50kDa
37kDa
20kDa
Na+/K+ ATPase
Tubulin
Flag
YKT6

Fig.S5

Fig.S5. (A) Ykt6 spectral counts from three independent experiments. N-terminal GFP-tagged fusion of WT Ykt6 or phosphomutants were immunoprecipitated with GFP and subjected to the mass spectrometry. **(B)** Representative western blot of GFP immunoprecipitations from HEK293T cells expressing either GFP, N-terminal GFP-tagged fusion of WT Ykt6 or phosphomutants. Cells were immunoblotted for GFP, STX17, and SNAP29. **(C)** Representative western blot of GFP immunoprecipitations from HEK293T cells expressing either GFP, N-terminal GFP-tagged fusion of WT Ykt6 or phosphomutants. Cells were immunoblotted for GFP and Gosr2. **(D)** Representative western blot of 3 after cell fractionation for endogenous Ykt6 from HEK293T cells transfected with the indicated Flag-tagged kinases and pharmacologically treated as in (S1D). Membrane fraction is defined by Na+K+ and cytosolic fraction is defined by Tubulin.

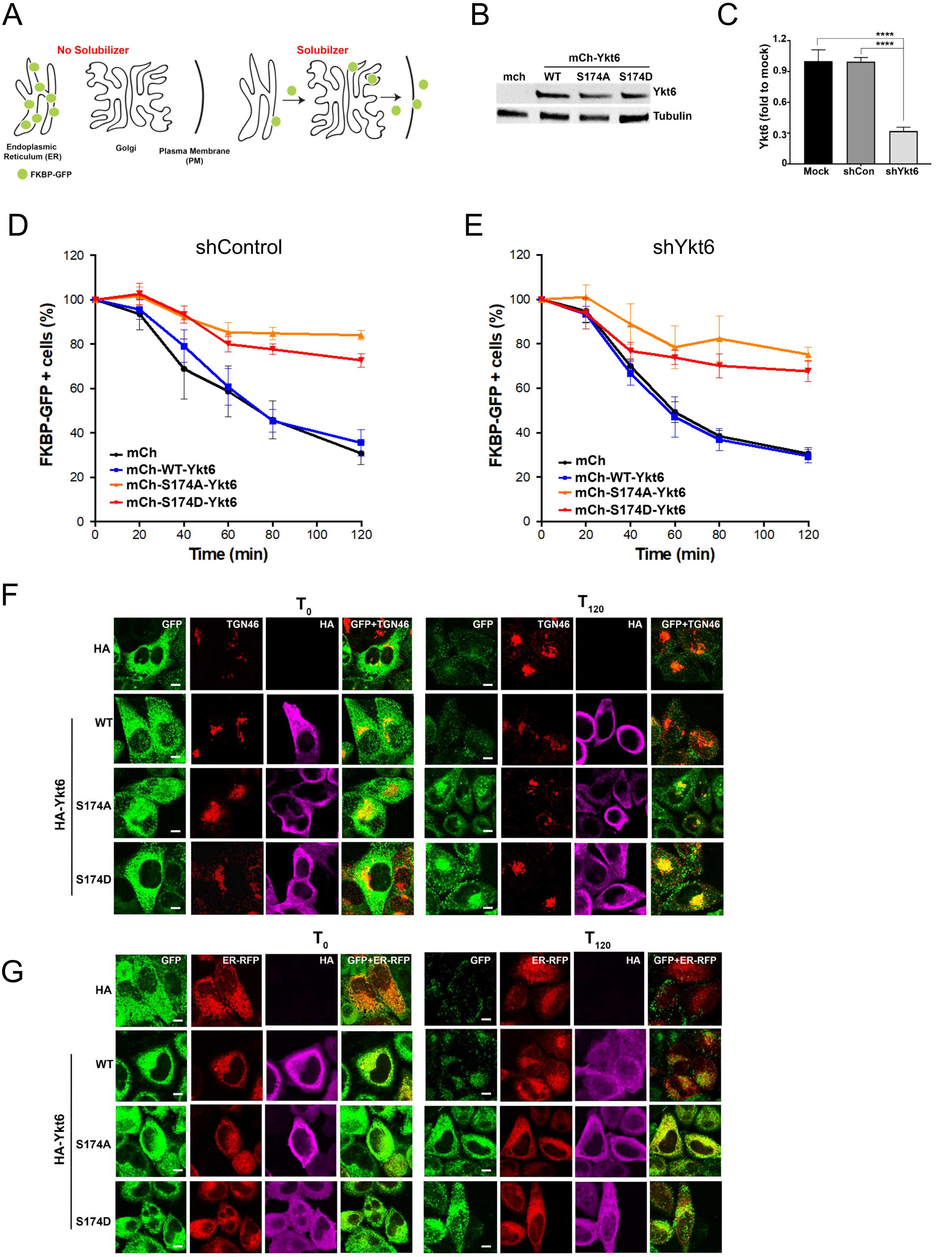

A
No Solubilizer
Solubilzer
Endoplasmic Reticulum (ER)
Golgi
Plasma Membrane (PM)
FKBP-GFP
B
mCh-Ykt6
mch
WT
S174A
S174D
Ykt6
Tubulin
C
Ykt6 (fold to mock)
****
****
Mock
shCon
shYkt6
D
shControl
FKBP-GFP + cells (%)
Time (min)
mCh
mCh-WT-Ykt6
mCh-S174A-Ykt6
mCh-S174D-Ykt6
E
shYkt6
FKBP-GFP + cells (%)
Time (min)
mCh
mCh-WT-Ykt6
mCh-S174A-Ykt6
mCh-S174D-Ykt6
F
T0
T120
GFP
TGN46
HA
GFP+TGN46
HA
WT
S174A
S174D
HA-Ykt6
G
T0
T120
GFP
ER-RFP
HA
GFP+ER-RFP
HA
WT
S174A
S174D
HA-Ykt6

Fig.S6

Fig.S6. **(A)** Mechanism of FKBP-GFP reporter to evaluate the progression of the secretory pathway in HeLa-derived PC4 cells. Diagram shows the movement of the reporter upon addition of the ligand/solubilizer. **(B)** Representative western blot of PC4 cell lysates overexpressing mCherry, N-terminal mCherry-tagged of WT Ykt6, phospho-ablative mutant (S174A) and phospho-mimetic mutant (S174D) of Ykt6 immunoblotted for Ykt6 (abcam). Tubulin served as loading control. **(C)** RT-qPCR analysis of endogenous levels of Ykt6 in PC4 cells treated with either control or Ykt6-specific shRNA. Values on Y-axis represent fold change of Ykt6 over mock treated cells. **(D-E)** FACS analysis depicting % FKBP-GFP-positive cells over time in PC4 cells treated with either shControl (E) or shYkt6 (F) followed by overexpression of mCherry, N-terminal mCherry-tagged of WT Ykt6, phospho-ablative mutant (S174A) and phospho-mimetic mutant (S174D) of Ykt6 post addition of solubilizer. **(F-G)** Representative immunofluorescence images of PC4 cells expressing FKBP-GFP reporter from figure 5 transiently transfected with HA-tagged WT Ykt6 or indicated phosphomutants of Ykt6 and immunostained with TGN-46 for trans-Golgi (F) or mCherry-Sec61 for the ER (G).

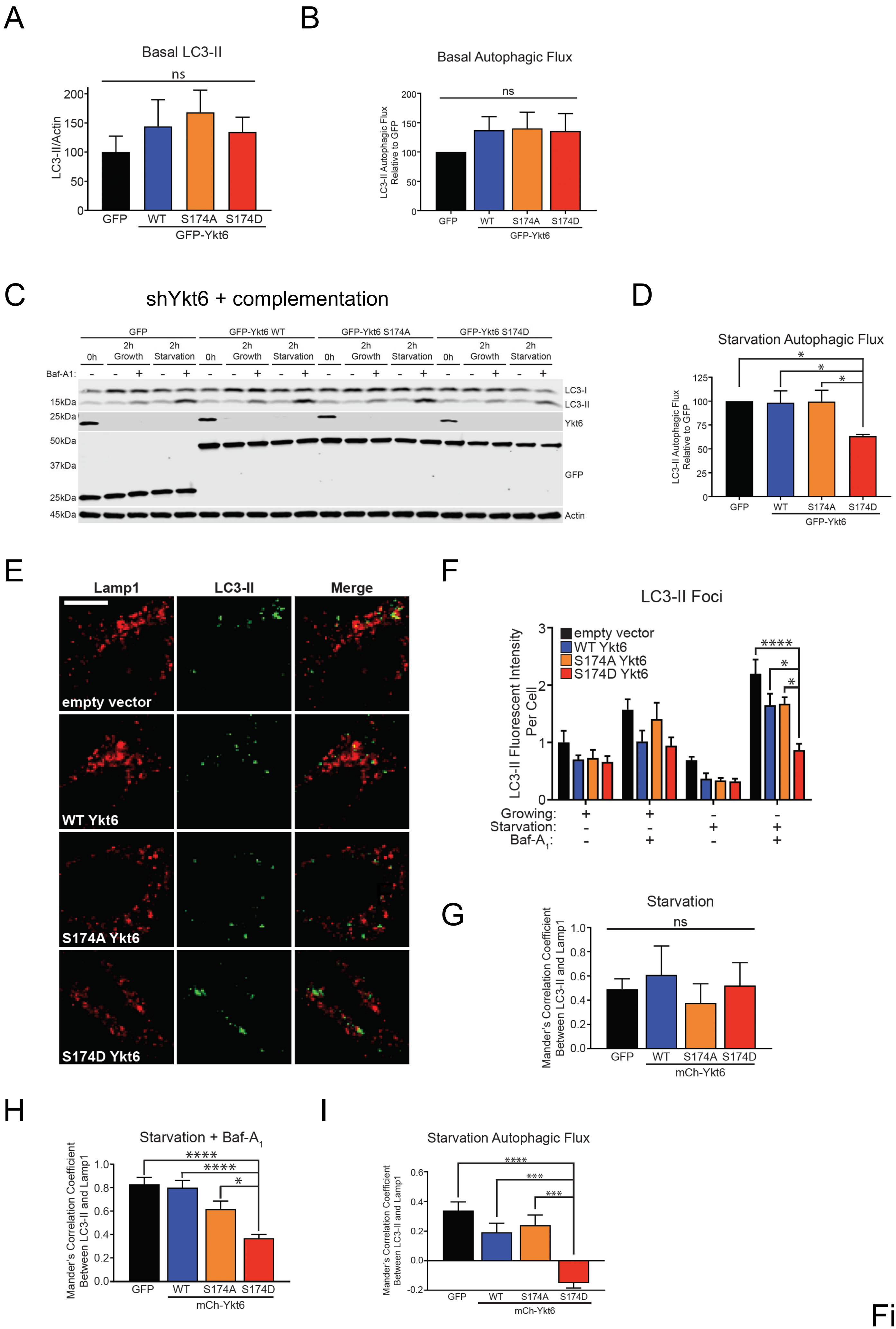

A
Basal LC3-II
ns
LC3-II/Actin
GFP
WT
S174A
S174D
GFP-Ykt6
B
Basal Autophagic Flux
ns
LC3-II Autophagic Flux Relative to GFP
C
shYkt6 + complementation
GFP
GFP-Ykt6 WT
GFP-Ykt6 S174A
GFP-Ykt6 S174D
0h
2h Growth
2h Starvation
Baf-A1:
LC3-I
LC3-II
Ykt6
GFP
Actin
15kDa
25kDa
50kDa
37kDa
45kDa
D
Starvation Autophagic Flux
E
Lamp1
LC3-II
Merge
empty vector
WT Ykt6
S174A Ykt6
S174D Ykt6
F
LC3-II Foci
LC3-II Fluorescent Intensity Per Cell
empty vector
WT Ykt6
S174A Ykt6
S174D Ykt6
Growing:
Starvation:
Baf-A1:
G
Starvation
Mander's Correlation Coefficient Between LC3-II and Lamp1
mCh-Ykt6
H
Starvation + Baf-A1
I
Starvation Autophagic Flux

Fig.S7

Fig.S7. **(A)** Densitometry analysis of basal LC3-II normalized to Actin from western blot Figure 5E. **(B)** Autophagic flux (defined as [LC3-II Baf-A_1] – [LC3-II]) from western blot in Figure 6A of growing conditions. N=6 *p<0.05 **p<0.01 ****p<0.0001 Two-way ANOVA, uncorrected Fisher's LSD test. **(C)** Representative western blot for LC3-II from a stably transfected PC12 cell line with a doxycycline inducible shRNA targeting endogenous rat Ykt6 and infected with lentiviruses carrying GFP and N-terminal GFP-tagged fusion of WT Ykt6 or phosphomutants. Cells were treated for 2 hours (2h) with 200nM Bafilomycin A_1 (Baf-A_1) in growing conditions (fresh 10% FBS and 4.5% glucose growth medium) or in starvation conditions (Torin-1 250nM, 1X HBSS supplemented with 10mM HEPES). Actin serves as a loading control. **(D)** Autophagic flux (defined as [LC3-II Baf-A_1] – [LC3-II]) from western blot in (C) of growing and starvation conditions. N=5 *p<0.05 **p<0.01 ****p<0.0001 One-way ANOVA, uncorrected Fisher's LSD test. **(E)** Representative immunofluorescence images of overexpressed GFP-LC3 and endogenous Lamp1 colocalization from HeLa cells expressing WT human Ykt6 and phosphomutants, under starvation conditions with Baf-A_1. **(F)** Normalized fluorescence intensity of GFP-LC3-II foci. **(G)** Colocalization analysis based on Mander's correlation coefficient between Lamp1 and LC3-II in starvation conditions from (E), starvation and **(H)** Baf-A_1 and **(I)** autophagic flux. N=3 *p<0.05 **p<0.01 ***p<0.001 ****p<0.0001 One-way ANOVA, Tukey's test. Scale bar is 5μm. (H)

A

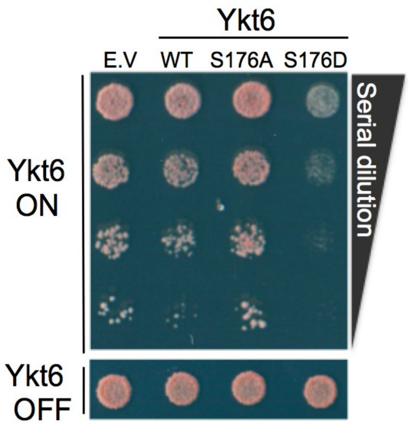
Ykt6
E.V
WT
S176A
S176D
Ykt6 ON
Ykt6 OFF
Serial dilution

B

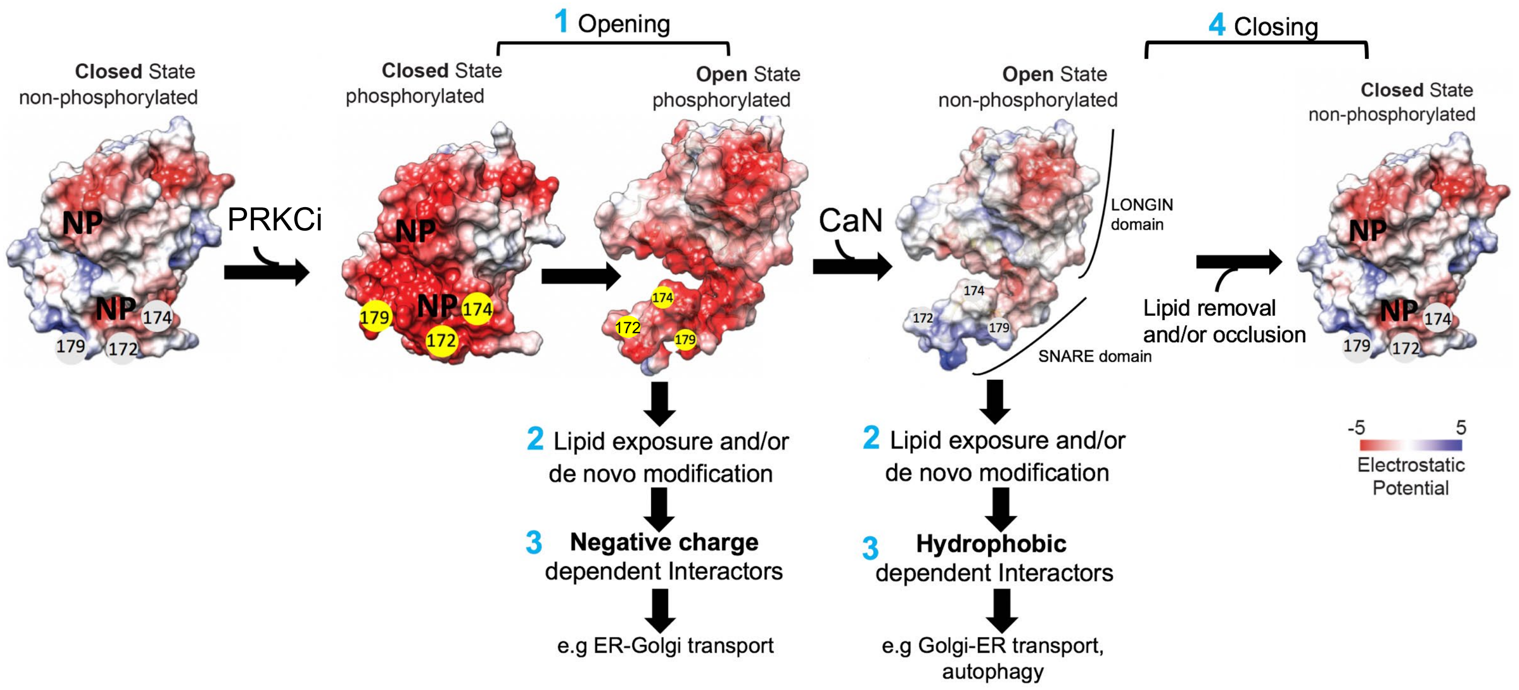
1 Opening
4 Closing
Closed State non-phosphorylated
Closed State phosphorylated
Open State phosphorylated
Open State non-phosphorylated
Closed State non-phosphorylated
PRKCi
CaN
NP
174
172
179
LONGIN domain
SNARE domain
Lipid removal and/or occlusion
2 Lipid exposure and/or de novo modification
3 Negative charge dependent Interactors
e.g ER-Golgi transport
2 Lipid exposure and/or de novo modification
3 Hydrophobic dependent Interactors
e.g Golgi-ER transport, autophagy
-5
5
Electrostatic Potential

Fig.S8

Fig.S8. (A) Yeast strains were spotted onto plates containing uninducing media [synthetic defined glucose (SD) −Leu; Gal-Ykt6 selective; *Lower*] and replica platted in four fold serial dilutions onto Ykt6–inducing plates containing selective media and (SGal −Leu) (*Upper*). Empty vector (EV) is used as control plasmid. Representative plate of n=3. **(B)** Surface model of Ykt6 closed conformation with the human CaN-sensitive phosphorylation sites highlighted: the yeast-animals evolutionarily conserved site S174 (174) and at the additional animal evolutionarily conserved CaN-dependent sites S172, T179 (172 and 179). NP=negative patch. Electrostatic potential computed and projected at the surface representation. Ykt6 exists in a closed conformation in the cytosol, phosphorylation of Ykt6 by PRKCi at the evolutionary-conserved CaN-sensitive site promotes its open conformation (step 1). Subsequently, either *de novo* lipid modification such as palmitoylation and/or complete exposure of farnesyl groups (step 2), would allow Ykt6 to be localized to membranes. Depending on the kinetics of phosphorylation, established by both kinase activity and or phosphatase activity such as CaN would dictate the affinity to its binding partners and ultimately the membrane compartment and vesicular function where Ykt6 would participate (step 3). Finally, dephosphorylation by CaN and potentially other phosphatases together with depalmitoylation would enable Ykt6 to return to its close conformation and cytosolic localization.

Dataset S1 (separate file). iTRAQ MS results from human WT GFP-Ykt6 immunoprecipitates from transfected HEK293T cells treated for 30 minutes with either vehicle (DMSO), the Ca^{2+} ionophore ionomycin (1μM), the calcineurin-specific inhibitor Tacrolimus (1μM) or co-treated with ionomycin and Tacrolimus.

Dataset S2 (separate file). MS results from GFP immunoprecipitates from HEK293T cells transfected with GFP alone, or N-GFP fusions of WT, S174A or S174D Ykt6. N=3 independent MS runs.

SI References

1 Gordon, D. E. et al. VAMP3/Syb and YKT6 are required for the fusion of constitutive secretory carriers with the plasma membrane. PLoS Genet 13, e1006698, (2017).

2 Sarbassov, D. D., Guertin, D. A., Ali, S. M. & Sabatini, D. M. Phosphorylation and regulation of Akt/PKB by the rictor-mTOR complex. Science 307, 1098-1101, (2005).

3 Taipale, M. et al. Quantitative analysis of HSP90-client interactions reveals principles of substrate recognition. Cell 150, 987-1001, (2012).

4 Kofoed, M. et al. An Updated Collection of Sequence Barcoded Temperature-Sensitive Alleles of Yeast Essential Genes. G3 (Bethesda) 5, 1879-1887, (2015).

5 Caraveo, G. et al. FKBP12 contributes to alpha-synuclein toxicity by regulating the calcineurin-dependent phosphoproteome. Proc Natl Acad Sci U S A 114, E11313-E11322, (2017).

6 Nguyen, M. & Krainc, D. LRRK2 phosphorylation of auxilin mediates synaptic defects in dopaminergic neurons from patients with Parkinson's disease. Proc Natl Acad Sci U S A 115, 5576-5581, (2018).

7 Smith, G. A., Gross, S. P. & Enquist, L. W. Herpesviruses use bidirectional fast-axonal transport to spread in sensory neurons. Proc Natl Acad Sci U S A 98, 3466-3470, (2001).

8 Schindelin, J. et al. Fiji: an open-source platform for biological-image analysis. Nat Methods 9, 676-682, (2012).

9 Williams, C. J. et al. MolProbity: More and better reference data for improved all-atom structure validation. Protein Sci 27, 293-315, (2018).

10 Pettersen, E. F. et al. UCSF Chimera--a visualization system for exploratory research and analysis. J Comput Chem 25, 1605-1612, (2004).

11 Crooks, G. E., Hon, G., Chandonia, J. M. & Brenner, S. E. WebLogo: a sequence logo generator. Genome Res 14, 1188-1190, (2004).

12 Baghirova, S., Hughes, B. G., Hendzel, M. J. & Schulz, R. Sequential fractionation and isolation of subcellular proteins from tissue or cultured cells. MethodsX 2, 440-445, (2015).

13 Ishihama, A., Shimada, T. & Yamazaki, Y. Transcription profile of Escherichia coli: genomic SELEX search for regulatory targets of transcription factors. Nucleic Acids Res, (2016).

14 Lescop, E., Schanda, P. & Brutscher, B. A set of BEST triple-resonance experiments for time-optimized protein resonance assignment. J Magn Reson 187, 163-169, (2007).

15 Delaglio, F. et al. NMRPipe: a multidimensional spectral processing system based on UNIX pipes. J Biomol NMR 6, 277-293, (1995).

16 Lee, W., Tonelli, M. & Markley, J. L. NMRFAM-SPARKY: enhanced software for biomolecular NMR spectroscopy. Bioinformatics 31, 1325-1327, (2015).

17 Shin, J., Lee, W. & Lee, W. Structural proteomics by NMR spectroscopy. Expert Rev Proteomics 5, 589-601, (2008).

18 Lee, W. et al. I-PINE web server: an integrative probabilistic NMR assignment system for proteins. J Biomol NMR 73, 213-222, (2019).
